# Exploring Stakeholder Perspectives on the Barriers and Facilitators of Implementing Digital Technologies for Heart Disease Diagnosis: Qualitative Study

**DOI:** 10.2196/66464

**Published:** 2025-03-05

**Authors:** Kamilla Abdullayev, Tim J A Chico, Jiana Canson, Matthew Mantelow, Oli Buckley, Joan Condell, Richard J Van Arkel, Vanessa Diaz-Zuccarini, Faith Matcham

**Affiliations:** 1 School of Psychology University of Sussex Falmer United Kingdom; 2 Clinical Medicine School of Medicine and Population Health The Medical School, University of Sheffield Sheffield United Kingdom; 3 School of Computing Engineering and Intelligent Systems Ulster University at Magee Londonderry United Kingdom; 4 Department of Computer Science Loughborough University Loughborough United Kingdom; 5 Department of Mechanical Engineering Imperial College London London United Kingdom; 6 Department of Mechanical Engineering University College London London United Kingdom

**Keywords:** heart disease, digital technologies, stakeholder perspectives, qualitative research, digital technology, health technology, heart, cardio, cardiology, cardiovascular, qualitative, focused group, quality of care, efficiency, digital health, mobile phone, artificial intelligence, AI

## Abstract

**Background:**

Digital technologies are increasingly being implemented in health care to improve the quality and efficiency of care for patients. However, the rapid adoption of health technologies over the last 5 years has failed to adequately consider patient and clinician needs, which results in ineffective implementation. There is also a lack of consideration for the differences between patient and clinician needs, resulting in overgeneralized approaches to the implementation and use of digital health technologies.

**Objective:**

This study aimed to explore barriers and facilitators of the implementation of digital technologies in the diagnosis of heart disease for both patients and clinicians, and to provide recommendations to increase the acceptability of novel health technologies.

**Methods:**

We recruited 32 participants from across the United Kingdom, including 23 (72%) individuals with lived experience of heart disease and 9 (28%) clinicians involved in diagnosing heart disease. Participants with experience of living with heart disease took part in semistructured focused groups, while clinicians contributed to one-to-one semistructured interviews. Inductive thematic analysis using a phenomenological approach was conducted to analyze the resulting qualitative data and to identify themes. Results were discussed with a cardiovascular patient advisory group to enhance the rigor of our interpretation of the data.

**Results:**

Emerging themes were separated into facilitators and barriers and categorized into resource-, technology-, and user-related themes. Resource-related barriers and facilitators related to concerns around increased clinician workload, the high cost of digital technologies, and systemic limitations within health care systems such as outdated equipment and limited support. Technology-related barriers and facilitators included themes related to reliability, accuracy, safety parameters, data security, ease of use, and personalization, all of which can impact engagement and trust with digital technologies. Finally, the most prominent themes were the user-related barriers and facilitators, which encompassed user attitudes, individual-level variation in preferences and capabilities, and impact on quality of health care experiences. This theme captured a wide variety of perspectives among the sample and revealed how patient and clinician attitudes and personal experiences substantially impact engagement with digital health technologies across the cardiovascular care pathway.

**Conclusions:**

Our findings highlight the importance of considering both patient and clinician needs and preferences when investigating the barriers and facilitators to effective implementation of digital health technologies. Facilitators to technology adoption include the need for cost-effective, accurate, reliable, and easy-to-use systems as well as adequate setup support and personalization to meet individual needs. Positive user attitudes, perceived improvement in care quality, and increased involvement in the care process also enhance engagement. While both clinicians and patients acknowledge the potential benefits of digital technologies, effective implementation hinges on addressing these barriers and leveraging facilitators to ensure that the technologies are perceived as useful, safe, and supportive of health care outcomes.

**International Registered Report Identifier (IRRID):**

RR2-10.1136/bmjopen-2023-072952

## Introduction

### Background

There has been a sharp rise in the use of digital health technologies in health care, particularly after the COVID-19 pandemic, which drove rapid adoption of remote measurement and consultation technologies [1-3]. In parallel, there has been a rapid growth in the use of consumer *well-being* devices marketed directly to citizens that monitor a range of health measures, such as sleep and heart rate [1-3]. Cardiovascular medicine has been one of the earliest adopters of digital technology in health care because aspects of cardiovascular health, such as electrocardiograms (ECGs), are already proven to be clinically relevant and are measurable using both medical devices and consumer wearables [4-6].

The potential benefits of using digital health technologies within cardiovascular health care are considerable, including early identification and modification of risk factors such as diabetes or hypertension; earlier, faster, or more accurate diagnosis; personalized treatment and management plans; improved ability to monitor disease and detect deterioration; and improved symptom assessment [7]. Meanwhile, health care systems are facing increasing challenges in delivering services designed in a predigital era. Existing care pathways remain rooted in face-to-face clinical assessments and siloed data about the patient across different analog and digital systems that are inaccessible to both the patient and their different care teams.

Digital health technologies could help address factors that contribute to delayed or inaccurate diagnosis of cardiovascular diseases [8]. An example of such an emerging technology is digital twins, which uses mathematical models to process data that are continuously updated to monitor various physiological symptoms over time [9-11]. This allows for the capture of longitudinal symptom data, provides customizable feedback for patients to help them alter behavior and self-manage their condition, and improves patient-clinician communication [12]. This efficient processing of large amounts of cardiovascular data highlights the substantial cost benefits of implementing digital health technologies [13].

The potential of digital technologies to improve health care has often been discussed, particularly by policy makers. However, it is also important to acknowledge that these novel technologies may pose risk, have negative effects on the users and the health care system, or face resistance from patients and clinicians. During the COVID-19 pandemic, patients reported several barriers to engagement with telehealth, including the lack of *human* contact, concerns related to confidentiality and data security, and a requirement for training in the use of new platforms [3]. Several qualitative studies have examined technology engagement among patients with cardiovascular diseases [14,15]. One recent review revealed 4 interrelated themes across 7 qualitative studies, including trust, safety and confidence, functionality and affordability, and risks and assurance, highlighting the complexity of factors contributing to patient engagement [14]. However, the focus of previous investigations has been primarily on technology used in rehabilitation or self-management of the confirmed disease [14,16-19]. However, the most common first stage of medical care is the diagnosis of symptoms that may reflect underlying heart disease, with an estimated 39% of adults experiencing symptoms that can reflect possible underlying heart disease such as chest pain [20]. Therefore, the initial onset of symptoms that may indicate cardiovascular problems affects a far greater number of people than those dealing with recovery from or management of heart disease. Furthermore, the diagnosis stage often comes with increased stress, frustration, and confusion for the patient and their families [21,22]. Thus, specific research is needed to understand the factors that influence the uptake of digital technologies at the stage of diagnosis, as these factors may differ from those that influence the use of technologies in people with proven heart disease.

Moreover, there is rarely a combined focus on both clinician and patient views, which prevents our ability to capture a more holistic perspective on the implementation of health care technology in clinical settings. Patients and clinicians have different needs and expectations of digital technologies, requiring specific exploration of approaches that can address these needs and expectations simultaneously. Al-Naher et al [23] examined factors influencing engagement in remote health care in heart failure and included both patient and clinician perspectives in their review. However, their final conclusions did not differentiate between these different user groups, applying the resulting 5 overarching themes (convenience, ease of use, education, clinical care, and communication) to both groups to provide insight to improve engagement [23], without adjustment based on user-specific needs. Meanwhile, 1 scoping review on the uptake of digital health technology across cardiovascular care provided separate barriers and facilitators between patient-level and clinician-level perspectives [24]. Their findings suggest that specific considerations should be made regarding user needs when attempting to implement acceptable and useful digital health technologies across different stages of cardiovascular care.

Ultimately, there remains a substantial gap in our understanding of the factors impacting engagement with digital health technologies for heart disease diagnosis across patients and clinicians. Therefore, more work is needed to provide stakeholder-led insights into specific barriers to target and facilitators to consider in the early stages of novel technology development, to improve engagement with, and thus the efficacy of, novel digital health technologies aiming to improve the accuracy and efficiency of heart disease diagnosis.

### Objectives

We used a qualitative approach to address the following objectives:

- Understand patients’ and clinicians’ views on the barriers and facilitators to the implementation of digital technologies for the diagnosis of heart disease
- Explore whether these perspectives on digital technology differ between patients and clinicians
- Provide evidence-based design considerations for novel digital health technologies to allow for more effective implementation for the diagnosis of heart disease

## Methods

### Overview

Our protocol and methodology have been previously published [25]. This study was conducted as part of a wider project aiming to test technologies available to diagnose a range of heart diseases and establish the most useful ways of communicating data back to clinicians and patients. The findings from this work have contributed to the development of testing priorities and procedures for a larger quantitative trial. The project represents a collaboration between clinical and research institutions across the United Kingdom.

The study was conducted and reported according to COREQ (Consolidated Criteria for Reporting Qualitative Research) [26] guidelines. The question topic guide involved 2 main parts: experiences relating to diagnostic delays and errors, and investigation of barriers and facilitators of engagement with technologies throughout the heart disease diagnosis pathway (Multimedia Appendix 1).

We have previously reported stakeholder experiences of heart disease diagnosis, specifically aiming to identify challenges contributing to delayed and inaccurate diagnosis [12]. This paper presents additional data collected to identify barriers and facilitators to the implementation of digital technologies for heart disease diagnosis, which are critical for uptake into clinical care.

### Study Design

A qualitative approach was taken to capture the depth and complexity of technology-related challenges faced by both patients and clinicians. We conducted semistructured focus groups with people with lived experience (LE) of heart disease to facilitate discussions on shared perspectives regarding the use of digital health technologies and to allow for direct comparisons among a range of diverse experiences with technology, which may have been missed in a one-on-one interview.

We conducted 1:1 interviews with clinicians to allow greater flexibility around their schedules and collect information across a range of clinical specialties.

### Patient and Public Involvement

All participant-facing materials were reviewed by a Sheffield-based cardiovascular patient advisory group. This ensured the information sheet, consent form, and focus group topic guides were accessible and easy to understand, including any technology-related terminology used. This led to the inclusion of a detailed description of the meaning of *digital*, followed by several examples of digital technologies throughout the questions covered.

### Study Population

Inclusion criteria for LE participants were a previous diagnosis of heart disease, aged ≥18 years, able to speak English sufficiently for participation, and able to consent to participate. Exclusion criteria included major cognitive impairment or dementia preventing participation. The inclusion criteria for clinicians were >6 months of experience in the diagnosis of heart disease, aged ≥18 years, able to speak English, and able to consent to participation.

The number of participants recruited for focus groups and interviews was based on pragmatic considerations [27], such as the time available for data collection against the wider project deadlines and the research team’s previous experience conducting qualitative research with clinicians [25]. With these practical considerations alongside recent evidence that data saturation can be achieved in as little as 9 interviews and 4 focus groups [28], we aimed to recruit between 4 and 6 LE participants across 4 focus groups to allow adequate time for each participant to share their views and experiences, and to interview 10 clinicians to achieve data saturation.

### Procedure

All participants were recruited in the United Kingdom, and data were collected between November 2022 and April 2023. We implemented a decentralized recruitment strategy, recruiting LE participants via Prolific (a web-based research platform), a panel for patients with cardiovascular diseases at the Sheffield University, and from UK-based participants from the Remote Assessment of Disease and Relapse–Major Depressive Disorder research study who had consented to be contacted for future research purposes [29]. Study information sheets were sent to people identified as meeting the eligibility criteria, with the advice to contact the study team if they were interested in participating. Study details were additionally shared on X, formerly known as Twitter. Individuals interested in participating were contacted via email to arrange an introductory phone call to confirm interest and eligibility. In this meeting, FM described the research and the procedure of the study. Recruitment materials can be found in Multimedia Appendix 2.

Clinicians were recruited using purposive sampling via personal and professional connections and a registered general physician Facebook (Meta Platforms, Inc) group. The study information sheets were posted on the Facebook group, with interested clinicians advised to contact the study team directly. Among them, clinicians represent a range of clinical roles across the heart disease pathway, from diagnosis through to long-term management. However, for the purposes of this study, we exclusively recruited those who diagnose heart disease on a regular basis. All information was given to clinicians via email before the web-based interview.

Consent and baseline demographic data were collected via web-based Qualtrics (Qualtrics International, Inc) surveys before qualitative data collection (Multimedia Appendix 3). The focus groups and interviews follow a preapproved, semistructured question schedule. Each focus group included either 5 or 6 participants. All focus groups and interviews were conducted on the web using Zoom (Zoom Video Communications), with focus groups lasting about 90 minutes and interviews ranging between 30 and 90 minutes, based on clinician availability. Interviews and focus groups were facilitated by KA, a psychology graduate working full time on the project. KA had no ongoing relationship with the participants and was not involved in their clinical care. She had neither previous experience in cardiology nor assumptions or expectations of the data. To support participants who may have found it challenging to engage with general questions about barriers and facilitators for digital technologies as a broad category, we included follow-up prompts and clarifying examples to help participants contextualize their responses, for instance, the provision of specific scenarios or requests to reflect on their experiences with technologies such as wearables, portable ECG monitors, or smartphones.

### Ethical Considerations

This study was reviewed and approved by the Sciences & Technology Cross-School Research Ethics Council at the University of Sussex (reference ER/FM409/1). It was conducted according to institutional and international guidelines for ethical research practices and complies with the Declaration of Helsinki regulations. Informed consent for each participant was acquired before data collection. Participants were provided with detailed information about the study objectives, procedures, and rights, including the right to withdraw at any time without penalty. The privacy and confidentiality of all participants was safeguarded through strict data protection measures. The focus group and interviews were audio recorded, anonymized, and then transcribed verbatim before analysis, with encryption and secure storage protocols implemented to prevent unauthorized data access. Field notes made during the focus groups were destroyed once transcripts were deidentified and finalized. Participants were compensated for their time with a £25 (US $31) Amazon voucher.

### Data Analysis

Data relating to patient and clinician perspectives on the facilitators and barriers of effective implementation of digital technologies into heart disease diagnosis were included in this analysis. Sample sociodemographic characteristics were also collected.

We conducted an inductive thematic analysis using a phenomenological approach, as this allowed us to be led by the data when exploring emerging themes related to stakeholder experiences. Our method was characteristic of a small q approach, as we followed the postpositivist framework of qualitative analysis to ensure the reliability of the resulting themes related to stakeholder experiences of heart disease diagnosis [30]. KA used NVivo (Lumivero) to conduct the first round of analysis, following the steps recommended by Braun and Clarke [31]. We used the 6-phase approach outlined by Braun and Clarke [31] to identify, analyze, and report patterns (themes) within the data. The six phases included the following: (1) familiarization with the data through reading and rereading, (2) generating initial codes, (3) searching for themes, (4) reviewing themes, (5) defining and naming themes, and (6) writing the report.

### Reflexivity and Positionality

To ensure methodological rigor, we adhered to the best practices outlined by Braun and Clarke [30], particularly focusing on avoiding common problems in thematic analysis, such as insufficient reflexivity or unclear connections between data and themes. In line with this updated guidance, we paid particular attention to how our own assumptions and positionalities might have influenced the analysis process. This reflexive approach was an integral part of our analysis, and we constantly questioned how our perspectives as researchers may have shaped the interpretation of the data.

We remained mindful of power dynamics, particularly during the clinician interviews and patient focus groups. Our familiarity with the clinical context and our personal experiences in conducting qualitative research shaped the way we interacted with participants and interpreted their responses. We also reflected on how the context of data collection (focus group vs individual interview) may influence the themes arising from the data and acknowledged and discussed these throughout the analysis process. This reflexive stance was crucial to ensure that we did not impose our own perspectives on the data, and we actively engaged in discussions with colleagues to challenge potential biases and enhance the trustworthiness of our findings.

### Scientific Rigor

We applied several strategies to ensure the trustworthiness of the study, addressing the dimensions of confirmability, dependability, credibility, and transferability.

To enhance confirmability, we maintained an audit trail throughout the study, documenting each step of the data collection and analysis process. This included detailed notes on our analytical decisions and the rationale for theme development. We ensured dependability by using a consistent approach to data collection, using semistructured interview guides, and by providing clear descriptions of the process of data analysis. Any deviations from the original plan were noted, and we made sure that the methods were applied systematically across all participants.

Credibility was enhanced through member checking, where we invited participants and other experts by experience to review and comment on the emerging findings. This process allowed us to verify our interpretations and ensure that they accurately represented participants’ experiences and perspectives. This was achieved through presenting the results of the first round of thematic analysis, which were presented to clinicians in the form of a research poster at the British Cardiology Society conference to increase the transferability of our results to a wider sample. A QR code was provided next to the poster, allowing clinicians to scan it and provide their reflections on whether we captured their experiences or comment on what was missing. Those unable to scan the code (eg, did not have a mobile available on hand) provided verbal feedback to the research poster presenter (KA). Feedback from 5 clinicians was integrated into the later stages of analysis.

We also consulted with a Sheffield-based cardiovascular patient advisory group again to provide further insight on the results of our analysis. Preliminary results were presented via a series of presentation slides summarizing the key themes that emerged. Verbal discussions were facilitated by the lead researcher (KA), and the meeting minutes were written up by JC.

## Results

### Sample Demographics

**Figure 1:**
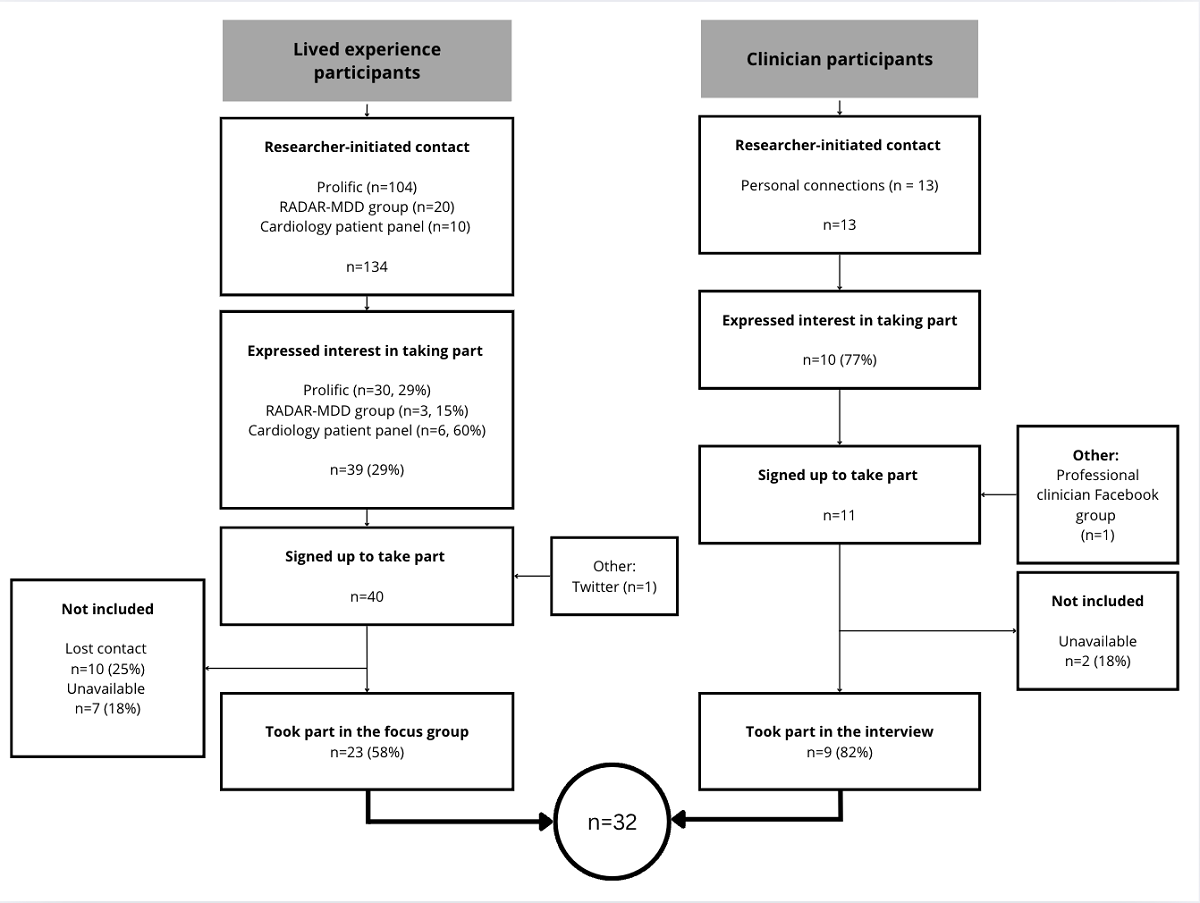
A flowchart of recruitment of participants, from initial contact to analysis. RADAR-MDD: Remote Assessment of Disease and Relapse–Major Depressive Disorder.

In total, 4 patient focus groups (n=23) and 9 individual clinician interviews were performed (n=32), shown in Figure 1. This represents 21.8% (32/147) of individuals initially contacted and 65% (32/49) of individuals who expressed initial interest in taking part. The sample of this study is reported in Table 1. This is the same group of participants that was used in the study by Abdullayev et al [12]; therefore, participants’ demographics are the same.

**Table 1:** Demographic characteristics of the sample (n=32).

Characteristic			Total sample (n=32)		LE^a^ participants (n=23)		Clinician (n=9)
Age (y), mean (SD; range)			58.0 (12.2; 31-76)		61.3 (11.5; 31-76)		48.5 (9.1; 35-60)
**Sex, n (%)**							
	Male	22 (69)		16 (70)		6 (67)	
	Female	10 (31)		7 (30)		3 (33)	
**Race and ethnicity, n (%)**							
	Asian	4 (12)		2 (9)		2 (22)	
	Black	0 (0)		0 (0)		0 (0)	
	White	27 (84)		21 (91)		6 (67)	
	Other (Arab)	1 (3)		0 (0)		1 (11)	
**Income bracket, n (%)**							
	<£15,000 (<US $18,800)	6 (19)		6 (26)		0 (0)	
	£15,000-£24,000 (US $18,800-US $30,200)	4 (12)		4 (17)		0 (0)	
	£24,000-£40,000 (US $30,200-US $50,300)	8 (25)		7 (30)		1 (11)	
	£40,000-£55,000 (US $50,300-US $69,200)	5 (16)		5 (22)		0 (0)	
	>£55,000 (>US $69,200)	7 (22)		1 (4)		6 (67)	
	Not disclosed	2 (6)		0 (0)		2 (22)	

^a^LE: lived experience.

Most clinicians (6/9, 67%) had been in practice for >20 years, representing primary (4/9, 44%), secondary (4/9, 44%), and emergency (1/9, 11%) care services. Most of the clinicians (8/9, 89%) reported feeling fairly to very confident using digital technologies, compared to 70% (16/23) of LE participants. All participants used at least these 3 devices: televisions, mobile phones, and laptops. The majority (27/32, 84%) also reported regularly using tablets or desktop computers. Table 1 summarizes the demographic and clinical characteristics of the sample.

### Analysis Results

Our analysis identified 6 themes arising from the participants’ views on digital technologies for the diagnosis of heart disease. A review of our efforts to increase the transferability of our findings via discussions with the Patient Advisory Board and clinicians attending a cardiology conference confirmed the value of considering both clinician and patient perspectives, as they felt this was key to implementing novel technology into health care. Insights provided by the advisory group reinforced confidence that our data fully captured the experience of stakeholders and resonated with their own LE.

Neither form of cross validation resulted in major changes to the analysis; however, it supported the organization and description of the themes and subthemes reported. While it is not possible to remove the subjective bias of the researchers conducting the analysis, this patient and public involvement–led approach to thematic analysis increases the credibility of our findings, which ultimately increases its transferability beyond our sample.

We organized these 6 themes into 2 key categories: barriers (defined as factors that prevent effective implementation) and facilitators (ways to enhance engagement among stakeholders). Textbox 1 summarizes the organization of the 6 themes that emerged from the data.

**Summary of the 6 themes emerging from the results of a thematic analysis with a phenomenological approach.**

**Themes and subthemes**

- Resource-related barriers: clinician workload, cost implications, and systemic barriers
- Technology-related barriers: complexity of technology, data security and privacy issues, safety concerns, and unreliability
- User-related barriers: negative user attitudes, worsening care experience, and individual-level variation
- Resource-related facilitators: cost-effectiveness, efficiency, and setup support
- Technology-related facilitators: accuracy and reliability, adequate safety considerations, ease of use, patients’ right to data, and personalization
- User-related facilitators: adapting to individual characteristics, positive user attitudes, and improving quality of care experience

### Theme 1: Resource-Related Barriers

#### Digital Technologies Can Add to Clinician Workload

Several clinicians raised considerable concerns regarding additional workload resulting from novel digital technologies being implemented into diagnosis. These participants emphasized that this would be a substantial barrier to the uptake of such health technologies given the current resource restraints within the National Health Service (NHS). Such concerns were not present among patient perspectives:

If it was going to make more work for me, if it was...to create any hassle for me I’m not interested.

Clinician8; male; aged 52 years

#### Digital Technologies Come With Cost Implications

Another resource-related barrier was the potential costs of digital technologies, both for the individual and the health care system. Clinicians highlighted current issues related to an imbalance between the cost versus benefits of collecting more patient health data and using it to improve patient health outcomes:

At best [they] had only marginal health, marginal impact but the cost of gathering the data and retrieving the important ones proved to be enormous.

Clinician1; male; aged 60 years

Patient perspectives also acknowledged how resource limitations within health care systems present challenges with implementing novel technologies in a sustainable way, as there appears to be a lack of connection between the development versus the implementation of digital health solutions:

That is what happens in the NHS. They all go off, do something, invent something and never do, they all come together because it costs billions of pounds to do it.

LE17; male; aged 65 years

#### Digital Technologies Are Not Immune to Systemic Barriers

Both clinicians and patients described how existing systemic barriers would prevent effective implementation due to a lack of access to appointments or equipment, a lack of support in initial setup, and difficulties integrating novel technologies into outdated NHS systems. Clinicians expressed doubt in their ability to support patients in setting up a device to aid with diagnosis within the limited appointment time they currently have:

GP appointments are 10 to 15 minutes, so how long is it going to take to explain this app, and how it works to them, and expect them to fill it in?

Clinician2; female; aged 38 years

Patients also shared frustrations with how outdated technology is within the NHS and how this inevitably acts as a barrier to the implementation of new technologies that could be used to improve heart disease diagnosis:

Sadly, the NHS is about 20 years behind with technology for a whole host of reasons.

LE17; male; aged 65 years

### Theme 2: Technology-Related Barriers

#### Complexity of Technology

The complexity of novel technology appears to be an important factor in engagement, as anything with too many steps or too many features to be learned will demotivate an individual’s engagement and produce inaccurate or incomplete data, which clinicians will not be able to use. Clinicians described how the complexity of a device will determine their willingness to engage with novel technologies:

I think how long or how easy or difficult it is to put or use this device, set it up and have it running and showing a patient what’s involved.

Clinician13; male; aged 49 years

Patients echoed these concerns, highlighting how increased complexity results in more errors within the data and prevents people from engaging with the device or program:

I think that the more complex it is, the more there is room for error, for a start, of actually producing the wrong data. And the second thing is that it may actually discourage people from using it.

LE29; male; aged 73 years

#### Issues With Data Security and Privacy

A key concern related to technology was the way sensitive health data would be protected. Clinicians reflected on potential issues that would arise if patients were not assured that their health data were being handled appropriately:

I can see some problems that include confidentiality, you know, these are personal information so you know we just have to make sure it’s very secure and you don’t know who has got access to this to this information.

Clinician7; male; aged 44 years

This concern was also seen among patient perspectives, with fears of large corporations having access to their health data acting as barriers to engaging in health technologies:

I’m not too sure whether they should be making money out of people’s illnesses or symptoms. I suppose it’s the data protection aspect of it.

LE4; male; aged 76 years

#### Concerns With Safety

Given the risks associated with monitoring symptoms before diagnosis, concerns related to the safety of the patient presented as an important barrier for both clinician and patient engagement. Clinicians emphasized the risks associated with collecting health data to monitor symptoms due to difficulties related to establishing safety parameters within the monitoring devices:

I think there is a governance issue about asking patients a question and then not processing safely the answer, to safety net them and the challenge there is getting the balance of safety versus being, you know, setting the threshold for seeking extra help to them and that’s where I think we’ve really struggled and never quite got it right.

Clinician8; male; aged 52 years

Moreover, patients expressed feelings of being unsafe in the case of emergency situations when their symptoms are being monitored remotely and doubt that health care staff would respond appropriately if their health was deemed at risk by the technology:

My worry about this is quite simple that the system would work but nobody would pick up on it, or actually do something about it if some if there was an emergency.

LE11; male; aged 70 years

#### Unreliability of Health Technologies

In addition to safety concerns, potential unreliability of a technology also emerged as a potential barrier to engagement. Clinicians described situations where they would be reluctant to depend on technology, as they do not feel confident in the reliability of the information it relays to the health care staff:

So to say to me, somebody’s got a heart attack when they haven’t, yeah, it’s massive. So I’m not suggesting that AI is doing that all the time, right, left, and centre. It’s definitely not doing that but it can do that.

Clinician1; male; aged 60 years

Similarly, patients shared doubts regarding how much they would be willing to rely on technological devices due to practical liabilities such as internet connection failure or poor connection in particular regions, as they fear it would pose a greater risk to their health compared to traditional approaches:

Another concern that comes to mind is how reliable it is in terms of the you know we’re all used to the internet going down like you lost your Internet connection, that could affect the technology used in this area. What happens if it all goes down, because what’s the back up? That’s a very valid concern.

LE19; male; aged 64 years

### Theme 3: User-Related Barriers

#### The Power of Negative User Attitudes

Negative attitudes toward the use of digital technology within health care were recognized as a potential barrier to engagement in several ways. First, distrust of technology providing reliable and useful information was evident among clinicians, highlighting how user attitudes might be influencing the way novel technologies are being implemented:

The blanket belief in AI is rubbish and AI can come up with rubbish if you are not careful.

Clinician1; male; aged 60 years

Meanwhile, another clinician felt that patients were more likely to possess this deep-rooted distrust in technology, suggesting there are still fears related to unethical health data collection, storage, and use:

Some of these conspiracy type theories where they think that what they’re being spied on.

Clinician12; male; aged 59 years

Some patients reflected that they would prefer not to have technology involved in the diagnosis pathway. They believed the health care system is implementing these novel systems to save money and do not care about how this impacts patient experiences and quality of care:

I just find it, it’s an extra barrier we’d rather not have, but because it’s cheap, and that doesn’t feel great to be treated in a cheap way, but that’s what it’s come down to, I think, which is very sad.

LE28; female; aged 50 years

Finally, a particularly influential user attitude is related to how useful or effective technology solutions were perceived to be. Both patients and clinicians reflected that they would not use a technology if they believed it was not going to benefit them or their patient. This highlights how refusing to engage in technology can be a rational decision made by the user, based on their personal beliefs regarding the potential utility:

There’s no point...if you get them to record stuff and cardiology don’t want it, and don’t look at it then actually they’re not going to use it.

Clinician2; female; aged 38 years

Why a chat bot when you can ring 111, and get the same advice from an actual living person?

LE5; female; aged 61 years

#### They Worsen Our Care Experience

Another barrier to engagement was the belief that the use of digital technology would worsen the quality of care. The burden of excessive interaction emerged as a potential barrier to engagement, as patients reflected on how frustration resulted in disengagement when patients are expected to dedicate a lot of their time to input data and track their symptoms:

I think the interactions got to be quite, quite minimal in a way because I think if you don’t, people will just not use you know they will get fed up, stop doing it.

LE29; male; aged 73 years

Moreover, excessive interaction may also result in increased anxiety among patients, as constantly monitoring and checking symptoms may exacerbate their condition and worsen their quality of life:

If I keep constantly checking that machine, then I’m going to, and it’s a little bit raised, or whatever I’m going to be continually worrying which doesn’t help your blood pressure.

LE5; female; aged 61 years

Clinicians shared this concern, expressing reluctance to recommend a technology that could potentially cause further harm or anxiety for their patients:

It may backfire because the patient might get the wrong idea might get panic, might get anxious you know it might they might think they are getting feedback, it must be something very severe you know. So those things can be a backfire, you know they might get upset. They might get anxious.

Clinician7; male; aged 44 years

Finally, there was a consistent message across both participant groups that digital technologies could never truly replace face-to-face human contact, and any attempts to do so will ultimately worsen the quality of care across the cardiovascular care pathway:

I don’t think you know a human face and a human voice will ever beat, you know will be beaten in the future. So I think you know we’ve got a struggle to do that, anyway.

LE8; male; aged 61 years

During COVID we found this because we thought, can we make use of some of these things? But what a lot of the patients said was missing actually was...more direct contact.

Clinician6; female; aged 49 years

#### There Is Too Much Individual-Level Variation

There was consistent acknowledgment of the challenges related to individual-level variation and how this would inevitably impact engagement with any digital health technology. It is clear that both patients and clinicians can have very different experiences, beliefs, and familiarity with digital technologies, and it is difficult to implement technologies that suit the needs of every potential user, especially given the variation across heart diseases.

One patient reflected on how their heart disease requires very different care compared to others, highlighting the challenges of implementing effective digital technology within different heart disease diagnosis pathways:

I’m not particularly into wearable devices, because I think that they’re probably far more useful for people who’ve got electrical problems with their heart, whereas mine is a plumbing issue, always has been.

LE10; male; aged 65 years

Clinicians also described how the nature of individual differences in preferences can act as a barrier to engagement, as it is not possible to suit everyone’s needs, especially when it comes to different demographic factors and previous experiences:

Some patients are going to be up for it, and they would love to have something on their phone and they like, you know, there are patients who really like to record data, and they will love it. They will get their phone, and they’ll get an app, and it will be fine. There are some who would be fairly resistant to it.

Clinician2; female; aged 38 years

Furthermore, clinicians expressed concerns regarding the accessibility of potential technologies, as any technology is heavily dependent on patients’ understanding of the device or program, which often varies but can be difficult to predict on a larger scale:

So you have an app that can help to monitor the condition but the patient couldn’t use it couldn’t put in the data, then there’s no point using those apps isn’t it?

Clinician7; male; aged 44 years

### Theme 4: Resource-Related Facilitators

#### It Needs to Be Cost-Effective

Clinicians considered evidence for the cost-effectiveness of a novel technology to be a facilitator of effective implementation; however, this was also dependent on adequate resources to support implementation from the relevant health care service or trust. This highlights the importance of considering financial implications from the costs to the individual to the costs to the health care system:

If it was going to be cost-effective you know, I don’t have any way of bringing in new technology the way my practice works currently, you know...but it needs to be some way of bringing staff in to help me do things like that.

Clinician13; male; aged 49 years

#### It Needs to Be Efficient

A key driver for engagement for both patients and clinicians related to the additional efficiency that health technologies could provide during the diagnosis process, as this could address current issues that are contributing to inaccurate or delayed heart disease diagnoses:

If it took the place of a 24-hour blood pressure monitoring or 24-hour ECG or what’s your average pulse over this time, then actually, that’s quite useful, because it’s kind of doing, taking away some of the work or putting the workload elsewhere. It’s doing the work that’s already being done.

Clinician2; female; aged 38 years

Patients also shared how increasing efficiency would improve the quality of their health care experience and therefore act as an important facilitator of their engagement with novel technologies:

The automation of the whole process is, would be a blessing for me.

LE10; male; aged 65 years

I suppose it could be, if it’s all digital data coming into one source that could be much more efficient.

LE28; female; aged 50 years

#### It Would Help to Have Setup Support

There was a shared sentiment between both patients and clinicians regarding the importance of having adequate setup support at the initial point of implementation of any digital technology. In particular, clinicians highlighted that as it is not feasible for them to provide this support due to current resource limitations, they would be comforted by the knowledge that there is an external body responsible for supporting patients to set up the technology, as well as providing adequate support in case of technological issues at any stage:

If there was like a support line, they could ring instead, then, you know, we could just direct, you know, and say, actually, that’s fine, or you will be contacted by the you know, this company will help you go through the app, then that’s fine, I suppose.

Clinician2; female; aged 38 years

Patients also reflected that adequate provision is needed to make people feel confident in engaging in any health technology related to their heart condition, with suggestions that language used in the setup support is crucial in increasing engagement among users:

I think you need somebody that’s gonna help you. You need very plain un-jargonistic instructions so that we can follow it

LE18; female; aged 66 years

### Theme 5: Technology-Related Facilitators

#### Is it Going to Be Accurate and Reliable?

Unsurprisingly, accuracy and reliability of technology were consistently brought up as important facilitators of engagement, as this elicits confidence in both clinicians and patients that they can use the technology to improve the quality of their experience or the accuracy of the diagnosis. Clinicians often expressed accuracy as the first thing they would consider when deciding whether to engage with a novel technology:

It should be accurate, I guess, accuracy is most important...good accuracy that would be ideal isn’t it? So most of the data can be interpreted by a machine

Clinician7; male; aged 44 years

This was consistently echoed by patients, who felt accuracy was the foundation of a good digital health solution and would only agree to use something they were confident would produce accurate data that could be used within their health care pathway:

It would need to be very accurate.

LE22; female; aged 68 years

It’s really hard to sort of summarize if you’re having seen a clinician...you need to summarize quite a few weeks worth of data...[technology] is far more accurate trying to get a snapshot from a from any from a patient about their overall health, and especially their mental health.

LE28; female; aged 50 years

#### Safety Has Been Adequately Considered

As mentioned previously, safety was a key area of discussion given the potential risks of monitoring symptoms before receiving a diagnosis. In fact, clinicians provided specific requirements for the way that data should be dealt with and thresholds that would need to be in place for them to feel confident in implementing novel technologies to aid in the diagnosis of heart diseases:

If it was kind of then inputting symptoms, it would have to have very strict criteria as to how it dealt with that. Yeah, I think, is the problem if it was just a manual thing that flashed up every time they entered, I have chest pain, you’re going to have to be very careful what it said or did.

Clinician2; female; aged 38 years

Moreover, patients also shared their perspective on how data should be shared safely among the device, the patient, and the clinician, highlighting the nuance in the communication of risk and potentially concerning health data collected by a digital device:

Anything which goes above a certain level of importance, it should go to the doctors or medics or emergency services as required, but it has to be quite, shall we say a severe level to actually get to the giving out that warning.

LE11; male; aged 70 years

#### Is it Easy for me to Use?

The consensus was that for any technology to be effectively implemented into clinical practice, it needs to be as simple as possible, as this produces the greatest level of widespread engagement and fewer complications for clinicians who need to use the data output:

Something that’s easy to use...convenient to use, you know, for everybody, for the patient and us. Because then I know that they’re more likely to use it.

Clinician6; female; aged 49 years

Patients also emphasized the importance of simplicity in novel technologies as well as making it easy to integrate them into current health care systems to ensure sustained engagement:

The key to get people to use anything is to make it easy. So, if we go down this route, which I think is great, we should piggy backing in on existing technologies...that can be used by every part of the NHS.

LE17; male; aged 65 years

#### Patients Have a Right to Their Data

There was considerable discussion surrounding who should have access to health data collected by digital devices aiding in the diagnosis pathway; however, general attitudes of participants suggested that patients have a right to their own data, regardless of what they are being monitored for, as this encourages trust between the patient and the clinician:

I mean yeah it should be sent to patients and I think lots of, because that’s the patient’s information at the end of the day, and I guess a lot about health care is being open and transparent and actually you shouldn’t be sending data out about a patient to the doctor and the patient not having that information.

Clinician2; female; aged 38 years

Interestingly, patients mainly expressed wanting clinicians to have access to their data, suggesting they did not feel confident in how to handle receiving their own health data without the support of a health care professional. This echoes previous concerns regarding safety and highlights the importance of making patients feel supported while depending on technology to collect and interpret their health data:

I would think the GP would be the first person to receive information and followed by myself and any associated to the medical profession, professional and in terms of when you refer to someone, a specialist, for example, if they’re already involved. So that’s the order that I would like to see it in.

LE19; male; aged 64 years

#### Personalization Is Key

When considering the development of health technologies, personalization was a key element mentioned as a facilitator of effective implementation. The clinicians’ shared perspective highlighted the importance of making people feel that the technology was tailored toward them, instead of expecting people to tailor themselves to the technology. There was also a sense that past experiences had led to high expectations of technology, placing greater pressure on developers to design health technologies that align with public perceptions:

But yeah, generally speaking, people like stuff that they feel isn’t just generic and sent out to everyone.

Clinician2; female; aged 38 years

Meanwhile, patients also emphasized the importance of receiving personalized and relevant data instead of generic feedback as a way of keeping people engaged. Patient perspectives also highlighted interest in examining trends and patterns within their health data, suggesting technologies should be designed based on the assumption that some patients may want to engage with their data beyond their clinical consultations:

What you’d want to do is to be able to interrogate the database that maybe there’s some graphs and trends to see. You know how your reading is compared to average.

LE10; male; aged 65 years

### Theme 6: User-Related Facilitators

#### Adapting to Individual Characteristics

Despite acknowledging how difficult it can be to develop health technologies tailored to individual differences, both patients and clinicians provided useful insights into how this could be done effectively to improve engagement. Clinicians emphasized the importance of asking patients how they wanted to interact with a digital technology as part of their diagnosis journey, as well as capturing clear expectations regarding their understanding and capabilities in relation to the technology as early as possible:

One way of addressing it is to ask the patient how much they would expect to interact. You know. That’s one way to it, you know to ask the patient.

Clinician7; male; aged 44 years

I think the patients understanding the technology and being able to use it and to use it appropriately.

Clinician9; male; aged 35 years

Meanwhile, patients reflected on the importance of considering the target demographic when designing any health technology, as well as increased difficulties resulting from comorbidities:

But let’s make it one device. So I don’t have to have all the other devices. Otherwise they’re going to be competing for my attention...I’m getting older and the target audience for this, most people who are ill are older, with multiple conditions.

LE17; male; aged 65 years

Overall, there was a clear message among participants that considering individual differences between patients is key to effective implementation and sustained engagement with novel health technologies aiming to improve heart disease diagnosis:

It also has to be, shall we say selective in what a single person or what the user requires it to do...so it has to be targeted individually to each individual person

LE11; male; aged 70 years

#### The Role of Positive User Attitudes

It seemed that individual attitudes toward technology more generally, as well as its use in health care, played an influential role in willingness to engage with novel health technologies. Both patients and clinicians expressed a very positive outlook on the value of incorporating technologies into heart disease diagnosis, which translated as a greater willingness to engage:

I think, to be honest, the NHS, we need to go more and more towards these apps

Clinician2; female; aged 38 years

A crucial facilitator was also a perception that the technology would in fact be useful for them, whether this was based on evidence to show it would improve an aspect of their care or if they judged it as being a helpful addition based on past experiences:

It needs to be proved. It needs to be shown to some degree that it’s definitely, it’s making, improving the outcome before I use it.

Clinician7; male; aged 44 years

Yeah, I think that’d be good to have like a chat bot, where if you’ve got any questions or anything like that, you can just click and get them answered rather than having to try and wait and get in to see the doctor or a consultant.

LE20; female; aged 54 years

However, there was still a recurring sentiment that complete dependence on technology is not feasible, with patients emphasizing the importance of human oversight even if data are being collected remotely. This highlights a key aspect of digitalized health care that is important to stakeholders and should be considered thoroughly during implementation to increase engagement and create a sense of safety among participants:

I think what should happen is that the medical profession should be getting the feedback and react accordingly to that.

LE29; male; aged 73 years

#### It Improves the Quality of Patient Care

Unsurprisingly, when stakeholders felt that they would experience direct benefits to the quality of their or their patients’ care, they felt more motivated to engage with novel technologies. There were specific benefits that were mentioned by participants, with some degree of variation between patients and clinicians. Patients reflected on past experiences with health technologies, which made their lives easier because it made handling health data more convenient:

Any digital technology is advantageous both to the user and supplier. And I’ll cite the Covid app, instead of carrying sheets and sheets of paper about with you if you go on holiday, on your Covid app, it tells you when you had it, where you had it, what it was that you got.

LE1; male; aged 72 years

Meanwhile, clinicians emphasized how having better access to their patients’ health data made their jobs easier and allowed for better quality of care that was adapted to both clinician and patient needs:

I can access patients’ information easier you know I don’t have to be in the on the ward. It’s just physically looking on the note, so it’s a lot of, improves the flexibility.

Clinician7; male; aged 44 years

An improved access to health data also reduced anxiety in patients, as they expressed a feeling of relief for themselves and their families because of feeling more informed about their condition or their symptoms:

It just gives you peace of mind. And obviously with your family members. They put the knowledge around them as well...So that’s it’s a no brainer really. It’s got to help.

LE8; male; aged 61 years

There was also evidence for a strong desire to be more involved in their own care pathway, as they felt this would improve their health care experiences and result in more transparency between the patient and the health care provider:

I would certainly welcome having more access to my medical records, because obviously, whenever I go and see a GP, I’m just amazed about how much data they’ve got about me, but I can’t see it. I wish I could.

LE10; male; aged 65 years

**Figure 2:**
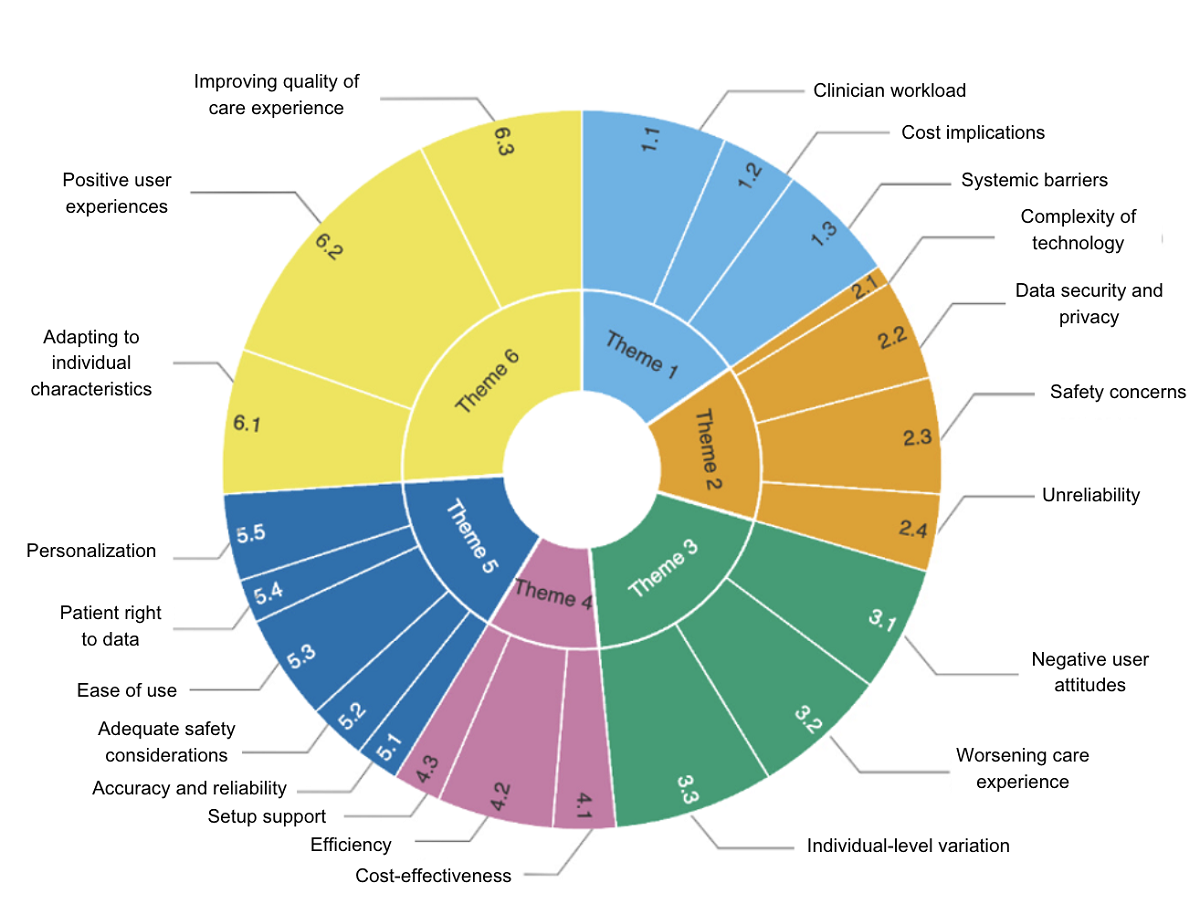
Sunburst visual of themes by size based on items coded, separated by themes, representing the barriers and facilitators of engagement with digital technologies for heart disease diagnosis.

Figure 2 presents the themes and subthemes described earlier in a sunburst diagram to illustrate the relative size of each subtheme within each of the 6 themes. This figure reveals that user-related barriers and facilitators (themes 3 and 6) emerged as the biggest themes, while resource-related barriers and facilitators (themes 1 and 4) were the smallest themes overall. Thus, these findings provide crucial insight to inform the development of novel health care technologies, particularly for the sake of making appropriate decisions to ensure user needs are met.

### Recommendations

On the basis of the emerging themes presented earlier, we have developed recommendations that should be considered when developing digital technologies to assist in the diagnosis of cardiovascular diseases. These recommendations are divided into technology-specific considerations (related to how the technologies function or are used) and system-level considerations (how the broader health care system should adapt to successfully implement such technologies). Multimedia Appendix 4 summarizes these recommendations based on each theme that came from the data, collected from participants with an interest in participating in digital technology research and clarified with support from the Patient Advisory Board.

## Discussion

### Principal Findings

This study has revealed the variety of barriers and facilitators influencing the effective implementation of digital technologies into the heart disease diagnosis pathway, as seen from the perspective of stakeholders with an interest in digital technology research. Both barriers and facilitators were organized into resource-, technology-, and user-related themes, with several subthemes within each of the 6 major themes.

Resource-related barriers and facilitators related to clinician workload, system-level influences, cost implications, efficiency, and support infrastructure. These findings are consistent with previous studies that have found increased clinician workload and a lack of integration into clinical workflow to be common barriers to the uptake of digital health technologies into cardiovascular care, while improved efficiency, institutional approval, and organizational support are all common facilitators [24,32]. Furthermore, technology-related barriers and facilitators included themes related to reliability, accuracy, safety parameters, data security, ease of use, and personalization. These perspectives were consistent with a recent qualitative review of wearable technology adoption for cardiac monitoring, which found 4 interrelated themes, including trust, safety and confidence, functionality and affordability, and risks and assurance [14]. Furthermore, concerns related to accessibility and usability of technology also emerged in a systematic review and content analysis of barriers and facilitators for health management across several physical and mental health conditions [33], highlighting the overlap in technology-related barriers among different stages of the care pathway. Overall, our findings emphasized key areas of technology development that could be adapted to improve the implementation of digital health technologies into the cardiovascular diagnosis pathway.

Finally, the most prominent themes were the user-related barriers and facilitators, which encompassed user attitudes, individual-level variations, and impact on quality of health care experiences. This theme captured a wide variety of perspectives among the sample and echoed findings from existing literature, which revealed how patient and clinician attitudes and personal experiences substantially impact engagement with digital health technologies across the cardiovascular care pathway, ranging from cardiac rehabilitation to remote care and self-management in heart failure [15,16,19,23]. These results also appear to be consistent across different clinical conditions, with a recent systematic review investigating barriers and facilitators to using digital health technologies finding that perceptions of usefulness and willingness to use novel technologies were important facilitators to enhance the uptake of digital health technologies by health care professionals across different clinical specialties [33]. Thus, the results of our study highlight the impact of user-related factors on the effective implementation of novel digital health technologies and therefore reveal a key area for future technology development to focus on to improve engagement levels during the diagnosis pathway.

Another key objective of this study was to understand potential differences between patient and clinician perspectives in relation to the barriers and facilitators mentioned earlier. Overall, the results of our study suggest that generally patients and clinicians share similar views on factors that may be preventing effective implementation of novel digital technologies into health care, as well as areas to focus on to facilitate better implementation. However, there were a few exceptions throughout the subthemes, with resource-related barriers (such as clinician workload and high costs) and technology-related safety concerns being discussed more by clinicians. Meanwhile, user-related barriers, such as negative attitudes toward technology and perceptions that quality of care would be reduced by novel technologies, were only presented as barriers by LE participants. These differences are consistent with the wider literature investigating factors influencing uptake of digital health technologies, as concerns related to resource restraints and evidence-based care also emerged as barriers in a sample of clinicians working with chronic obstructive pulmonary disease [34,35]. Moreover, while facilitators were mostly similar between both participant groups, the only exceptions were resource-related cost benefits and technology-related accuracy and reliability, which were facilitators emphasized by clinicians.

It is not surprising that clinicians presented more resource- and technology-related perspectives given they are more likely to be exposed to these aspects of novel technologies compared to patients [36]. It is also expected that patient perspectives would focus more on user experience and impact on quality of care, as they are able to draw on personal LE of how digital technologies used in their own care impacted their experiences. This distinction is consistent with the review by Whitelaw et al [24], which found that increased workload and a lack of integration with electronic medical records were identified as clinician-level barriers, while organizational support and improving efficiency were important facilitators according to clinician perspectives. A scoping review [32] focusing on hypertension management also found that concerns with integration of technologies into existing clinical workflow only emerged among health care professionals, while interference with patient- health care provider relationships was primarily a patient concern. Ultimately, our data highlight how different user groups may vary in which barriers are more influential in preventing them from engaging with health technologies within the heart disease diagnosis pathway. Therefore, the findings of this study provide useful insights into how implementation processes can be tailored to target these specific barriers, as well as consider facilitators, to increase uptake of novel health technologies within the heart disease diagnosis pathway.

The recommendations based on our qualitative findings for implementing health care technologies focused on addressing resource, technology, and user-related factors. Key strategies include integrating intuitive interfaces with existing IT systems, providing comprehensive training and support, and ensuring cost-effective models. Addressing technology-related barriers involves designing user-friendly, secure, and reliable systems with rigorous clinical trials and active monitoring for issues. Simplifying complexity and ensuring transparent data use are also essential. Facilitators for successful implementation include demonstrating cost-effectiveness, improving efficiency, and offering extensive setup support for patients and clinicians. Ensuring accuracy and reliability through rigorous validation and regulatory frameworks, alongside enabling patient access to their data, is vital. Emphasizing personalization and adapting to individual user characteristics will further enhance user acceptance and improve the overall care experience. These considerations echo existing calls to address key issues associated with implementing technologies into clinical care, such as ensuring patients can trust the systems managing their data and clinicians are not overwhelmed by the large volume of data that are generated by wearable digital health technologies [37]. However, while these general recommendations provide a foundation, they may lack specificity when applied to certain contexts. For example, the type of heart diseases targeted by a digital diagnostic tool will influence not only its design but also its adoption and integration into existing care pathways. Similarly, the demographic and clinical characteristics of patients using the device, such as age, literacy, and comorbidities, may present unique challenges that require tailored solutions [38]. Finally, while the focus on cost-effectiveness and efficiency is commendable, these factors must be balanced against equity considerations. For example, ensuring access to these technologies for underserved populations or regions with limited resources is critical to avoid widening existing health care disparities. Therefore, a nuanced approach that considers these broader contextual, systemic, and equity-focused challenges is essential for the successful implementation of health care technologies [39].

### Strengths and Limitations

A key strength of this study was the use of a qualitative study design to capture both patient and clinician experiences. This depth of insight would not have been possible to achieve using quantitative methods. The use of a decentralized recruitment strategy for both participant groups also meant our sample included people from across the country and captured a range of health care and technology experiences. Moreover, patient and public involvement was intentionally incorporated into each stage of the study, from the creation of study materials to the review of preliminary thematic analysis results. This increases confidence that the study’s design effectively created a comfortable environment for participants to share their experiences and ensured their data were interpreted accurately. While it is not possible to remove subjective bias from the lead researcher’s interpretation and analysis of the qualitative data, the involvement of patient panels and LE advisers throughout the study can provide reassurance that the results are translatable beyond our sample.

However, there are several limitations that also need to be acknowledged. The web-based nature of our recruitment method may have resulted in a biased sample of individuals who were more confident using technology, meaning their experiences are unlikely to capture the challenges faced by patients and clinicians who have less experience with technologies. Moreover, we were not successful in recruiting *difficult to reach groups*, such as ethnic minority groups with different cultural experiences across the United Kingdom, despite efforts to use the research team’s personal connections to include participants from underserved communities. This would have been extremely valuable to aid in our understanding of challenges related to accessibility and implementation of novel health technologies, so we suggest future research studies attempt to build on our findings and explore perspectives on barriers and facilitators in populations that are more resistant, or less experienced, in using digital health technologies. Our exclusion of people who were not fluent in English means our results exclude perspectives from people who may face different challenges and benefits from interacting with technology. An additional consideration is the differing forms of data collection. We made the pragmatic decision to run focus groups with LE participants and individual interviews with clinicians, due to the difficulties in getting multiple clinicians to be free at the same time for a focus group. This difference in data collection methods may have influenced results. Focus groups can result in more dynamic exchanges and can help foster a shared understanding of a phenomenon, resulting in different information shared than would be in an individual scenario. In contrast, interviews can allow for deeper, more personal insights to be shared [40]. While there is some precedent for the combination of qualitative methods, with researchers suggesting that it can be a useful method of triangulation to enhance depth and breadth of insights [41], there is ongoing debate about how different data collection methods can be most meaningfully combined in analysis. While we attempted to address this with our reflective approach to analysis, it is possible that our results and key findings may have differed if the same qualitative methods had been used to collect data from both LE and clinician participants.

Although the questions asked in focus groups and interviews were designed to be as vague and nonleading as possible, it should be acknowledged that this study was conducted as a part of a wider project aiming to develop a novel digital twin technology to improve holistic heart disease diagnosis. This meant the topic guides for both focus groups and interviews were focused on a specific technology being designed for a specific purpose; thus, it is possible that this may have excluded experiences and perspectives on other potential technologies that could be used within the heart disease diagnosis pathway.

Finally, we did not specifically recruit participants with direct experience of using digital technologies for health management. This intentional choice aimed to broaden the applicability of our findings; however, it may have impacted the nature of participants’ responses, introducing a degree of hypothetical reasoning. However, even without direct experience of using these technologies or implementing them in health care services, all participants brought valuable insights based on their LEs with health care services, use of technologies in daily lives, and existing challenges in the system. Analytically, we handled this challenge by carefully interpreting the data within the scope of participants’ experiences and triangulating results across multiple participants and sources to ensure that conclusions were not drawn from speculative responses.

### Conclusions

Digital technologies are a growing area, and our results provide insight into the key design and implementation characteristics needed to be accepted by patients and clinicians into routine clinical care. This qualitative study has revealed the multifaceted barriers and facilitators influencing the implementation of digital technologies in the heart disease diagnosis pathway. The findings demonstrate that resource-, technology-, and user-related factors play critical roles in adoption, with user-related aspects emerging as particularly important. While patients and clinicians generally share similar perspectives on implementation challenges and opportunities, notable differences exist in their prioritization of specific barriers and facilitators. These insights emphasize the importance of tailored implementation strategies that address the unique concerns of both user groups. To increase the acceptability of novel health technologies in heart disease diagnosis, future developments should prioritize creating user-friendly, secure, and reliable systems that can be integrated into existing clinical infrastructure, as well as allowing for personalization and adaptability to individual user needs. Addressing these factors is key to fostering confidence in and uptake of digital diagnostic tools in cardiovascular care.

## Appendix

Multimedia Appendix 1 Question schedule.

Multimedia Appendix 2 Information sheets and consent forms.

Multimedia Appendix 3 Participant demographics questionnaire.

Multimedia Appendix 4 Themes and subthemes.

## Abbreviations

COREQ: Consolidated Criteria for Reporting Qualitative Research
ECG: electrocardiogram
LE: lived experience
NHS: National Health Service

## Data Availability

The datasets generated or analyzed during this study are available from the corresponding author on reasonable request.
