# Supplementary material for: Exploring Stakeholder Perspectives on the Barriers and Facilitators of Implementing Digital Technologies for Heart Disease Diagnosis: Qualitative Study
Source: JMIR Cardio. 2025 Mar 5;9:e66464. doi: 10.2196/66464 (PMC11923470; doi:10.2196/66464)
Supplement: Multimedia Appendix 2 [file cardio_v9i1e66464_app2.pdf]

UNIVERSITY OF SUSSEX

STUDY TITLE:
Improving Holistic Community-based Diagnosis of Heart Disease: Interviews with Individuals with Clinicians with experience of diagnosing Heart Disease

INVITATION TO TAKE PART:

We would like to invite you to take part in a research study. Before you decide whether you want to take part, it is important for you to understand why the research is being done and what it would involve for you. Please take time to read the following information carefully and discuss it with others if you wish.

This information sheet tells you the purpose of this study, what will happen if you take part, and gives you detailed information about the conduct of the study. Ask us if there is anything that is not clear or if you would like more information.

WHAT IS THE PURPOSE OF THE STUDY?

Today, accurate diagnosis of heart disease depends almost entirely on how the patient describes their symptoms to a non-specialist GP. Mobile phones, wearables such as smartwatches, and other technologies are now very common and can obtain a lot of information that could help diagnose heart disease faster and more accurately. However, translation into clinical settings requires us to understand how patients and doctors might use the technologies and understand the data they provide.

This project will work with patients and clinicians to learn what information they believe is necessary to diagnose a range of heart diseases. We will develop and test the technologies required to obtain this information. We will then develop ways to communicate this data to both patients and clinicians to provide the greatest possible insights into people's physical and mental health.

WHY HAVE I BEEN INVITED TO TAKE PART IN THIS STUDY?

We are asking healthcare professionals involved in the diagnosis of heart disease to take part in this study. This includes (but is not limited to) primary and secondary care physicians, nurses, and allied healthcare professionals). Clinicians must have at least 6 months of experience working with heart disease patients; be aged 18 or over; and be able to speak fluent English.

DO I HAVE TO TAKE PART?

Taking part in this study is completely voluntary. You can take as long as you wish to decide whether you want to take part. If you decide to participate, we will ask you to sign a consent form to show you have agreed and will keep a copy. You are free to withdraw from the study at any time, without having to give a reason, and this will not affect any aspect of your work.

WHAT WILL HAPPEN TO ME IF I TAKE PART?

We will arrange a time for a one-on-one interview with you over Zoom using a University of Sussex account, which will run for a maximum of 2 hours. You will receive £25 for your participation. The first 30 minutes will be devoted to answering any questions about the study, signing the online consent form and completing forms about background information about yourself. We will securely store an electronic copy of this consent form on password-protected devices. The remaining time will be a discussion with the interviewer which will give you a chance to express your opinions on the topics this study aims to investigate. You do not have to answer any questions you do not wish to, and any responses will remain confidential. These sessions will be audio recorded, and you will notice that one of the researchers will be guiding the conversation while the other will be mostly taking notes. A few weeks after the session, the researchers will be in touch again to send you the results from the discussion. This will be an opportunity for you to corroborate whether your views are adequately represented.

After this initial interview, you will be asked to participate in another one-to-one interview with the researcher, which will last a maximum of 1-hour and you will be paid a further £15. This will be arranged for a future date, convenient for you. During this interview, you will be shown some examples of how data collected via digital technologies may be visualised to clinicians and asked to provide feedback about them. You can decide not to participate in the second interview without it affecting your participation in the first interview.

On occasion, we may have undergraduate Psychology students observing interviews as part of their undergraduate research project. You will be notified of this in advance and have the opportunity to request that students are <u>not</u> present in your interview if you would prefer.

WHAT ARE THE POSSIBLE BENEFITS OF TAKING PART?

Although there is the possibility that the study may not directly benefit you, we hope you will find the discussion interesting. If you do decide to take part, you will be contributing towards research which aims to improve holistic community-based diagnosis of heart disease using advanced digital health tools.

WHAT ARE THE POSSIBLE DISADVANTAGES AND RISKS OF TAKING PART?

There are no anticipated risks of taking part in this study. Though unlikely, it is possible that the discussion may induce distressing thoughts or feelings. An experienced mental health researcher will be conducting the interview, under the close supervision of a qualified Health Psychologist. The researchers will be able to guide the discussion and check levels of comfort with the topics discussed throughout the interview.

WHAT WILL HAPPEN IF I DON'T WANT TO CARRY ON WITH THE STUDY?

If you withdraw from the study, we will keep the information about you that we have already obtained. To safeguard your rights, we will use the minimum personally identifiable information possible. If you choose to withdraw once the discussion has already begun, you will still be given the £25.

WILL MY INFORMATION IN THIS STUDY BE KEPT CONFIDENTIAL?

Anything that is discussed in the interview, will be treated confidentially. All information collected about you will be kept strictly private and stored securely on appropriate servers. This project involves the use of a University of Sussex Zoom account. Details of the platform's privacy notice can be found here: Zoom Privacy Policy. All data collected will be stored securely on a University of Sussex managed system. Only members of the research team will have access to your personal data. The only exception to this is if you tell us something which may cause concern for risk of serious harm or death to yourself or others. In this case, we may have to inform the appropriate services, but we will always communicate this to you first.

At the beginning of the study, you will be asked to create a unique identifier code that will be used to identify all information we keep about you. Your name, address and other identifiable information about you, will be kept in a separate place so that it will not be possible to identify any data stored about you. All audio recordings will be destroyed after they have been transcribed and checked for accuracy. Any reports or publications arising from this study will be anonymised and will not include your name or any details that could lead to someone learning your identity outside of the study site. All the requirements of the data protection legislation will apply to the processing of your personal data.

If you would like more information about how your data will be processed under the terms of UK data protection laws please visit the link below: https://www.sussex.ac.uk/ogs/policies/information/dpa

WHAT SHOULD I DO IF I WANT TO TAKE PART?
If after reading the information sheet you would like to participate in the study, you can email the lead researcher Kamilla Abdullayev (kga21@sussex.ac.uk) to confirm your availability. Before starting the online interview, you will be asked to fill out an online survey to give full informed consent and you will not be able to take part in the study until you have done so.

WHAT WILL HAPPEN TO THE RESULTS OF THE RESEARCH STUDY?

The results of this study will be passed on to a team of engineers who will create a prototype of a holistic digital monitoring tool to help improve diagnosis of heart disease. The findings will all be anonymised but will collectively inform which features will be included in this device and how it can be effectively implemented into clinical care. The ultimate goal is to improve how heart disease is diagnosed, making it faster and more accurate. We also plan to publish the findings collected from all the clinician interviews to contribute to the cardiovascular health literature by providing a clinician perspective on the diagnostic process. If you would like to receive updates about papers or other activities which arise from this research, let us know in the consent form and we will notify you. Personal data will be destroyed within 10 years of collection; however the anonymised findings of the interviews will be securely stored for future research at the University of Sussex.

WHO IS FUNDING THE RESEARCH?

This project is being carried out by researchers and undergraduate students at the University of Sussex, School of Psychology and has been funded by the Engineering and Physical Sciences Research Council (EPSRC).

WHO HAS REVIEWED THE STUDY?

This research has been approved by Sciences & Technology Cross-Schools Research Ethics Committee (SCITEC) at the University of Sussex. The ethical review application number of this study is ER/FM409/2.

CONTACT DETAILS

If you have any concerns relating to this project please contact lead researcher, Kamilla Abdullayev (kga21@sussex.ac.uk) or chief investigator Dr Faith Matcham (f.matcham@sussex.ac.uk) and/or the Chair of the Science and Technology Cross Schools Research Ethics Committee, (crecscitec@sussex.ac.uk.

INSURANCE

The University of Sussex has insurance in place to cover its legal liabilities in respect of this study.

THANK YOU

Thank you for taking the time to read this information sheet carefully before giving consent to participate in this study.

DATE

31st October 2022

STUDY TITLE:
Improving Holistic Community-based Diagnosis of Heart Disease: Focus Groups with Individuals with Lived Experience of Heart Disease

INVITATION TO TAKE PART:

We would like to invite you to take part in a research study. Before you decide whether you want to take part, it is important for you to understand why the research is being done and what it would involve for you. Please take time to read the following information carefully and discuss it with others if you wish.

This information sheet tells you the purpose of this study, what will happen if you take part, and gives you detailed information about the conduct of the study. Ask us if there is anything that is not clear or if you would like more information.

WHAT IS THE PURPOSE OF THE STUDY?

Today, accurate diagnosis of heart disease depends almost entirely on how the patient describes their symptoms to a non-specialist GP. Mobile phones, wearables such as smartwatches, and other technologies are now very common and can obtain a lot of information that could help diagnose heart disease faster and more accurately. However, translation into clinical settings requires us to understand how patients and doctors might use the technologies and understand the data they provide.

This project will work with patients and clinicians to learn what information they believe is necessary to diagnose a range of heart diseases. We will develop and test the technologies required to obtain this information. We will then develop ways to communicate this data to both patients and clinicians to provide the greatest possible insights into people's physical and mental health.

WHY HAVE I BEEN INVITED TO TAKE PART IN THIS STUDY?

We are asking people to take part in this study if they are aged 18 or over, able to speak fluent English, and have lived experience of heart disease, whether that is in the past or present. You do not have to be currently undergoing NHS treatment to be eligible to participate.

DO I HAVE TO TAKE PART?

Taking part in this study is completely voluntary. You can take as long as you wish to decide whether you want to take part. If you decide to participate, we will ask you to sign a consent form to show you have agreed and will keep a copy. You are free to withdraw from the study at any time, without having to give a reason, and this will not reflect negatively on you in any way.

WHAT WILL HAPPEN TO ME IF I TAKE PART?

We will arrange a time for you to come to the University of Sussex campus for a group discussion. We will also conduct an online focus group for those who are not able to attend in person, using a University of Sussex Zoom account. The group will be moderated by two experienced researchers, and there will be approximately 5 other participants, this will run for a maximum of 2 hours. You will receive £25 for your participation and if the focus group is held in-person you will receive reimbursement for your travel.

The first 30 minutes will be devoted to answering any questions about the study, signing the online consent form and completing forms about background information about yourself. We will securely store an electronic copy of this consent form on password-protected devices. The remaining time will be a group discussion where everyone will have a chance to express their opinions on the topics this study aims to investigate. You do not have to answer any questions you do not wish to, and any responses will remain confidential. These sessions will be audio recorded, and you will notice that one of the researchers will be guiding the conversation while the other will be mostly taking notes. A few weeks after the session, the researchers will be in touch again to send you the results from the discussion. This will be an opportunity for you to corroborate whether your views are adequately represented.

After this focus group, you will be asked to participate in another one-to-one interview with the researcher, which will last a maximum of 1-hour and you will be paid a further £15. This will be arranged for a future date, convenient for you. During this interview, you will be shown some wearable devices and examples of data visualisations and be asked to provide feedback about them. You can decide not to participate in the interview without it affecting your participation in the focus group.

On occasion, we may have undergraduate Psychology students observing the focus groups and interviews as part of their undergraduate research project. You will be notified of this in advance and have the opportunity to request that students are not present in your focus group or interview if you would prefer.

WHAT ARE THE POSSIBLE BENEFITS OF TAKING PART?

Although there is the possibility that the study may not directly benefit you, we hope you will find the discussion with your peers interesting. If you do decide to take part, you will be contributing towards research which aims to improve holistic community-based diagnosis of heart disease using advanced digital health tools.

WHAT ARE THE POSSIBLE DISADVANTAGES AND RISKS OF TAKING PART?

There are no anticipated risks of taking part in this study. Though unlikely, it is possible that the discussion may induce distressing thoughts or feelings. An experienced mental health researcher will be conducting the focus group, under the close supervision of a qualified Health Psychologist. The researchers will be able to guide the discussion and check levels of comfort with the topics discussed throughout the group.

WHAT WILL HAPPEN IF I DON'T WANT TO CARRY ON WITH THE STUDY?

If you withdraw from the study, we will keep the information about you that we have already obtained. To safeguard your rights, we will use the minimum personally identifiable information possible. If you withdraw before the group discussion begins, all your transport costs will still be covered. If you withdraw once the discussion has already begun, you will still be given the £25.

Because of the nature of focus groups, it is not possible for us to withdraw your data once it has been given – there are usually lots of people talking, and it can be hard to pick out who is saying what, and what one person says may have an effect on what another participant says. Therefore, if you do choose to withdraw from this study then the researchers will retain any data that you have provided up to that point and will include this data in any subsequent analyses.

WILL MY INFORMATION IN THIS STUDY BE KEPT CONFIDENTIAL?

Anything that is discussed in the group will stay in the group – we will ask you to please respect people's privacy by not sharing any details of what was discussed. All information collected about you will be kept strictly private and stored securely on appropriate servers.
This project involves the use of a University of Sussex Zoom account. Details of the platform's privacy notice can be found here: Zoom Privacy Policy. All data collected will be stored securely on a University of Sussex managed system. Only members of the research team will have access to your personal data. The only exception to this is if you tell us something which may cause concern for risk of serious harm or death to yourself or others. In this case, we may have to inform the appropriate services, but we will always communicate this to you first.

At the beginning of the study, you will be asked to create a unique identifier code that will be used to identify all information we keep about you. Your name, address and other identifiable information about you, will be kept in a separate place so that it will not be possible to identify any data stored about you. All audio recordings will be destroyed after they have been transcribed and checked for accuracy. Any reports or publications arising from this study will be anonymised and will not include your name or any details that could lead to someone learning your identity outside of the study site. All the requirements of the data protection legislation will apply to the processing of your personal data.

If you would like more information about how your data will be processed under the terms of UK data protection laws please visit the link below: https://www.sussex.ac.uk/ogs/policies/information/dpa

WHAT SHOULD I DO IF I WANT TO TAKE PART?

If after reading the information sheet you would like to participate in the study, you can email the lead researcher Kamilla Abdullayev (kga21@sussex.ac.uk) to confirm your availability. Before starting the focus group, you will be asked to fill out an online survey to give full informed consent and you will not be able to take part in the study until you have done so.

WHAT WILL HAPPEN TO THE RESULTS OF THE RESEARCH STUDY?

The results of this study will be passed on to a team of engineers who will create a prototype of a holistic digital monitoring tool to help improve diagnosis of heart disease. The findings will all be anonymised but will collectively inform which features will be included in this device and how it can be effectively implemented into clinical care. The ultimate goal is to improve how heart disease is diagnosed, making it faster and more accurate. We also plan to publish the findings collected from all the focus groups to contribute to the cardiovascular health literature by providing a patient perspective on the diagnostic process. If you would like to receive updates about papers or other activities which arise from this research, let us know in the consent form and we will notify you. Personal data will be destroyed within 10 years of collection; however the anonymised findings of the interviews will be securely stored for future research at the University of Sussex.

WHO IS FUNDING THE RESEARCH?

This project is being carried out by researchers and undergraduate students at the University of Sussex, School of Psychology and has been funded by the Engineering and Physical Sciences Research Council (EPSRC).

WHO HAS REVIEWED THE STUDY?

This research has been approved by Sciences & Technology Cross-Schools Research Ethics Committee (SCITEC) at the University of Sussex. The ethical review application number of this study is ER/FM409/2.

CONTACT DETAILS

If you have any concerns relating to this project please contact lead researcher, Kamilla Abdullayev (kga21@sussex.ac.uk) or chief investigator Dr Faith Matcham (f.matcham@sussex.ac.uk) and/or the Chair of the Science and Technology Cross Schools Research Ethics Committee, (crecscitec@sussex.ac.uk.

INSURANCE
The University of Sussex has insurance in place to cover its legal liabilities in respect of this study.

THANK YOU
Thank you for taking the time to read this information sheet carefully before giving consent to participate in this study.

DATE
31st October 2022

Digital Twin Consent Form Interview

Start of Block: info sheet

Q17 Project Title: A Digital Twin to Improve Holistic Community-based Diagnosis of Heart Disease
Lead Researcher: Kamilla Abdullayev, School of Psychology
C-REC Ref no: ER/FM409/2

Q18 Would you like to re-read the information sheet you were previously sent?

- ○ Yes please (1)
- ○ No thank you, I have already read it (2)

Page Break

Display This Question:

If Would you like to re-read the information sheet you were previously sent? = Yes please

Q22 STUDY TITLE: Improving Holistic Community-based Diagnosis of Heart Disease: Interviews with Individuals with Clinicians with experience of diagnosing Heart Disease

INVITATION TO TAKE PART: We would like to invite you to take part in a research study. Before you decide whether you want to take part, it is important for you to understand why the research is being done and what it would involve for you. Please take time to read the following information carefully and discuss it with others if you wish. This information sheet tells you the purpose of this study, what will happen if you take part, and gives you detailed information about the conduct of the study. Ask us if there is anything that is not clear or if you would like more information.

WHAT IS THE PURPOSE OF THE STUDY? Today, accurate diagnosis of heart disease depends almost entirely on how the patient describes their symptoms to a non-specialist GP. Mobile phones, wearables such as smartwatches, and other technologies are now very common and can obtain a lot of information that could help diagnose heart disease faster and more accurately. However, translation into clinical settings requires us to understand how patients and doctors might use the technologies and understand the data they provide. This project will work with patients and clinicians to learn what information they believe is necessary to diagnose a range of heart diseases. We will develop and test the technologies required to obtain this information. We will then develop ways to communicate this data to both patients and clinicians to provide the greatest possible insights into people's physical and mental health.

WHY HAVE I BEEN INVITED TO TAKE PART IN THIS STUDY? We are asking healthcare professionals involved in the diagnosis of heart disease to take part in this study. This includes (but is not limited to) primary and secondary care physicians, nurses, and allied healthcare professionals). Clinicians must have at least 6 months of experience working with heart disease patients; be aged 18 or over; and be able to speak fluent English.

DO I HAVE TO TAKE PART? Taking part in this study is completely voluntary. You can take as long as you wish to decide whether you want to take part. If you decide to participate, we will ask you to sign a consent form to show you have agreed and will keep a copy. You are free to withdraw from the study at any time, without having to give a reason, and this will not affect any aspect of your work.

WHAT WILL HAPPEN TO ME IF I TAKE PART? We will arrange a time for a one-on-one interview with you over Zoom using a University of Sussex account, which will run for a maximum of 2 hours. You will receive £25 for your participation. The first 30 minutes will be devoted to answering any questions about the study, signing the online consent form and completing forms about background information about yourself. We will securely store an electronic copy of this consent form on password-protected devices. The remaining time will be a discussion with the interviewer which will give you a chance to express your opinions on the

topics this study aims to investigate. You do not have to answer any questions you do not wish to, and any responses will remain confidential. These sessions will be audio recorded, and you will notice that one of the researchers will be guiding the conversation while the other will be mostly taking notes. A few weeks after the session, the researchers will be in touch again to send you the results from the discussion. This will be an opportunity for you to corroborate whether your views are adequately represented. After this initial interview, you will be asked to participate in another one-to-one interview with the researcher, which will last a maximum of 1-hour and you will be paid a further £15. This will be arranged for a future date, convenient for you. During this interview, you will be shown some examples of how data collected via digital technologies may be visualised to clinicians and asked to provide feedback about them. You can decide not to participate in the second interview without it affecting your participation in the first interview. On occasion, we may have undergraduate Psychology students observing interviews as part of their undergraduate research project. You will be notified of this in advance and have the opportunity to request that students are not present in your interview if you would prefer.

WHAT ARE THE POSSIBLE BENEFITS OF TAKING PART? Although there is the possibility that the study may not directly benefit you, we hope you will find the discussion interesting. If you do decide to take part, you will be contributing towards research which aims to improve holistic community-based diagnosis of heart disease using advanced digital health tools.

WHAT ARE THE POSSIBLE DISADVANTAGES AND RISKS OF TAKING PART? There are no anticipated risks of taking part in this study. Though unlikely, it is possible that the discussion may induce distressing thoughts or feelings. An experienced mental health researcher will be conducting the interview, under the close supervision of a qualified Health Psychologist. The researchers will be able to guide the discussion and check levels of comfort with the topics discussed throughout the interview.

WHAT WILL HAPPEN IF I DON'T WANT TO CARRY ON WITH THE STUDY? If you withdraw from the study, we will keep the information about you that we have already obtained. To safeguard your rights, we will use the minimum personally identifiable information possible. If you choose to withdraw once the discussion has already begun, you will still be given the £25.

WILL MY INFORMATION IN THIS STUDY BE KEPT CONFIDENTIAL? Anything that is discussed in the interview, will be treated confidentially. All information collected about you will be kept strictly private and stored securely on appropriate servers. This project involves the use of a University of Sussex Zoom account. Details of the platform's privacy notice can be found here: [Zoom Privacy Policy]. All data collected will be stored securely on a University of Sussex managed system. Only members of the research team will have access to your personal data. The only exception to this is if you tell us something which may cause concern for risk of serious harm or death to yourself or others. In this case, we may have to inform the appropriate services, but we will always communicate this to you first. At the beginning of the study, you will be asked to create a unique identifier code that will be used to identify all information we keep about you. Your name, address and other identifiable information about you, will be kept in a

separate place so that it will not be possible to identify any data stored about you. All audio recordings will be destroyed after they have been transcribed and checked for accuracy. Any reports or publications arising from this study will be anonymised and will not include your name or any details that could lead to someone learning your identity outside of the study site. All the requirements of the [data protection legislation](#) will apply to the processing of your personal data. If you would like more information about how your data will be processed under the terms of UK data protection laws please visit the link below:
https://www.sussex.ac.uk/ogs/policies/information/dpa

WHAT SHOULD I DO IF I WANT TO TAKE PART? If after reading the information sheet you would like to participate in the study, you can email the lead researcher Kamilla Abdullayev (kga21@sussex.ac.uk) to confirm your availability. Before starting the online interview, you will be asked to fill out an online survey to give full informed consent and you will not be able to take part in the study until you have done so.

WHAT WILL HAPPEN TO THE RESULTS OF THE RESEARCH STUDY? The results of this study will be passed on to a team of engineers who will create a prototype of a holistic digital monitoring tool to help improve diagnosis of heart disease. The findings will all be anonymised but will collectively inform which features will be included in this device and how it can be effectively implemented into clinical care. The ultimate goal is to improve how heart disease is diagnosed, making it faster and more accurate. We also plan to publish the findings collected from all the interviews to contribute to the cardiovascular health literature by providing a clinician perspective on the diagnostic process. If you would like to receive updates about papers or other activities which arise from this research, let us know in the consent form and we will notify you. Personal data will be destroyed within 10 years of collection, however the anonymised findings of the interviews will be securely stored for future research at the University of Sussex.

WHO IS FUNDING THE RESEARCH? This project is being carried out by researchers and undergraduate students at the University of Sussex, School of Psychology and has been funded by the Engineering and Physical Sciences Research Council (EPSRC).

WHO HAS REVIEWED THE STUDY? This research has been approved by Sciences & Technology Cross-Schools Research Ethics Committee (SCITEC) at the University of Sussex. The ethical review application number of this study is ER/FM409/2

CONTACT DETAILS: If you have any concerns relating to this project please contact lead researcher, Kamilla Abdullayev (kga21@sussex.ac.uk) or chief investigator Dr Faith Matcham (f.matcham@sussex.ac.uk) and/or the Chair of the Science and Technology Cross Schools Research Ethics Committee, (crecscitec@sussex.ac.uk.

INSURANCE: The University of Sussex has insurance in place to cover its legal liabilities in respect of this study.

THANK YOU: Thank you for taking the time to read this information sheet carefully before

giving consent to participate in this study.

DATE: 31st October 2022

End of Block: info sheet

Start of Block: consent info

Q0 The following questions will be asking you to give your informed consent to take part in the study. In order to participate in the study, you will need to answer 'YES' to all of the questions below. Please read each question carefully and ask the researcher any questions if you are unsure. You have the right to withdraw at any point, participation is entirely voluntary.

End of Block: consent info

Start of Block: questions

*

Q1 Please enter your unique identifier code (Last three letters of your last name followed by the last three digits of your phone number). Please do not enter any spaces or capital letters. It should look similar to this: abc123

____

Q2 I consent to being interviewed by the researcher.

- ○ YES (1)

Q3 I consent to being interviewed on-line using the University of Sussex Zoom account.

- ○ YES (1)

Q4 I understand that my participation is entirely voluntary, that I can choose to withdraw from the interview at any point without having to give a reason and without penalty

○ YES (1)

Q5 I understand I can request without penalty that my data be withdrawn and deleted even after the interview, up until the data is analysed (estimated August 2023).

○ YES (1)

Q6 I understand that any information I provide is confidential, and that no information that I disclose will lead to the identification of any individual in the reports on the project, either by the researcher or by any other party.

○ YES (1)

Q7 I understand that my personal data will be used for the purposes of this research study and will be handled in accordance with Data Protection legislation. I understand that the University's Privacy Notice provides further information on how the University uses personal data in its research.

○ YES (1)

Q23 I understand that my collected data will be stored in a de-identified way (e.g. using ID numbers not names), and kept separate from other details about me (e.g. from the consent form). Electronic data will be stored securely on a University managed system

○ YES (1)

Q24 I understand that de-identified data may be made publicly available, for example through Open Science Framework online data repositories, journal publication or at the request of other researchers.

○ YES (1)

Q25 I have read the information sheet, had the opportunity to ask questions and I understand the principles, procedures and possible risks involved.

○ YES (1)

Q8 (Optional) I agree to be contacted by members of this team about future research that may stem from this study.

○ YES (1)

○ NO (2)

Q26 (Optional) I would like to be updated with the results of the study.

○ YES (1)

○ NO (2)

Q9 I agree to take part in the above University of Sussex research project

○ YES (1)

End of Block: questions

Digital Twin Consent Form Focus Group

Start of Block: ppt type and info sheet

Q17 Project Title:A Digital Twin to Improve Holistic Community-based Diagnosis of Heart Disease
Lead Researcher: Kamilla Abdullayev, School of Psychology
C-REC Ref no: ER/FM409/2

Q18 Would you like to re-read the information sheet you were previously sent?

- ◯ Yes please (1)
- ◯ No thank you, I have already read it (2)

Page Break

Display This Question:

If Would you like to re-read the information sheet you were previously sent? = Yes please

Q19 STUDY TITLE: Improving Holistic Community-based Diagnosis of Heart Disease: Focus Groups with Individuals with Lived Experience of Heart Disease

INVITATION TO TAKE PART: We would like to invite you to take part in a research study. Before you decide whether you want to take part, it is important for you to understand why the research is being done and what it would involve for you. Please take time to read the following information carefully and discuss it with others if you wish. This information sheet tells you the purpose of this study, what will happen if you take part, and gives you detailed information about the conduct of the study. Ask us if there is anything that is not clear or if you would like more information.

WHAT IS THE PURPOSE OF THE STUDY? Today, accurate diagnosis of heart disease depends almost entirely on how the patient describes their symptoms to a non-specialist GP. Mobile phones, wearables such as smartwatches, and other technologies are now very common and can obtain a lot of information that could help diagnose heart disease faster and more accurately. However, translation into clinical settings requires us to understand how patients and doctors might use the technologies and understand the data they provide. This project will work with patients and clinicians to learn what information they believe is necessary to diagnose a range of heart diseases. We will develop and test the technologies required to obtain this information. We will then develop ways to communicate this data to both patients and clinicians to provide the greatest possible insights into people's physical and mental health.

WHY HAVE I BEEN INVITED TO TAKE PART IN THIS STUDY? We are asking people to take part in this study if they are aged 18 or over, able to speak fluent English, and have lived experience of heart disease, whether that is in the past or present. You do not have to be currently undergoing NHS treatment to be eligible to participate.

DO I HAVE TO TAKE PART? Taking part in this study is completely voluntary. You can take as long as you wish to decide whether you want to take part. If you decide to participate, we will ask you to sign a consent form to show you have agreed and will keep a copy. You are free to withdraw from the study at any time, without having to give a reason, and this will not reflect negatively on you in any way.

WHAT WILL HAPPEN TO ME IF I TAKE PART? We will arrange a time for you to come to the University of Sussex campus for a group discussion. We will also conduct an online focus group for those who are not able to attend in person, using a University of Sussex Zoom account. The group will be moderated by two experienced researchers, and there will be approximately 5 other participants, this will run for a maximum of 2 hours. You will receive £25 for your participation and if the focus group is held in-person you will receive reimbursement for your travel. The first 30 minutes will be devoted to answering any questions about the study, signing the online consent form and completing forms about background information about yourself. We

will securely store an electronic copy of this consent form on password-protected devices. The remaining time will be a group discussion where everyone will have a chance to express their opinions on the topics this study aims to investigate. You do not have to answer any questions you do not wish to, and any responses will remain confidential. These sessions will be audio recorded, and you will notice that one of the researchers will be guiding the conversation while the other will be mostly taking notes. A few weeks after the session, the researchers will be in touch again to send you the results from the discussion. This will be an opportunity for you to corroborate whether your views are adequately represented. After this focus group, you will be asked to participate in another one-to-one interview with the researcher, which will last a maximum of 1-hour and you will be paid a further £15. This will be arranged for a future date, convenient for you. During this interview, you will be shown some wearable devices and examples of data visualisations and be asked to provide feedback about them. You can decide not to participate in the interview without it affecting your participation in the focus group. On occasion, we may have undergraduate Psychology students observing the focus groups and interviews as part of their undergraduate research project. You will be notified of this in advance and have the opportunity to request that students are not present in your focus group or interview if you would prefer.

WHAT ARE THE POSSIBLE BENEFITS OF TAKING PART? Although there is the possibility that the study may not directly benefit you, we hope you will find the discussion with your peers interesting. If you do decide to take part, you will be contributing towards research which aims to improve holistic community-based diagnosis of heart disease using advanced digital health tools.

WHAT ARE THE POSSIBLE DISADVANTAGES AND RISKS OF TAKING PART? There are no anticipated risks of taking part in this study. Though unlikely, it is possible that the discussion may induce distressing thoughts or feelings. An experienced mental health researcher will be conducting the interview, under the close supervision of a qualified Health Psychologist. The researchers will be able to guide the discussion and check levels of comfort with the topics discussed throughout the group.

WHAT WILL HAPPEN IF I DON'T WANT TO CARRY ON WITH THE STUDY? If you withdraw from the study, we will keep the information about you that we have already obtained. To safeguard your rights, we will use the minimum personally identifiable information possible. If you withdraw before the group discussion begins, all your transport costs will still be covered. If you withdraw once the discussion has already begun, you will still be given the £25. Because of the nature of focus groups, it is not possible for us to withdraw your data once it has been given – there are usually lots of people talking, and it can be hard to pick out who is saying what, and what one person says may have an effect on what another participant says. Therefore, if you do choose to withdraw from this study then the researchers will retain any data that you have provided up to that point and will include this data in any subsequent analyses.

WILL MY INFORMATION IN THIS STUDY BE KEPT CONFIDENTIAL? Anything that is discussed in the group will stay in the group – we will ask you to please respect people's privacy

by not sharing any details of what was discussed. All information collected about you will be kept strictly private and stored securely on appropriate servers. This project involves the use of a University of Sussex Zoom account. Details of the platform's privacy notice can be found here: Zoom Privacy Policy. All data collected will be stored securely on a University of Sussex managed system. Only members of the research team will have access to your personal data. The only exception to this is if you tell us something which may cause concern for risk of serious harm or death to yourself or others. In this case, we may have to inform the appropriate services, but we will always communicate this to you first. At the beginning of the study, you will be asked to create a unique identifier code that will be used to identify all information we keep about you. Your name, address and other identifiable information about you, will be kept in a separate place so that it will not be possible to identify any data stored about you. All audio recordings will be destroyed after they have been transcribed and checked for accuracy. Any reports or publications arising from this study will be anonymised and will not include your name or any details that could lead to someone learning your identity outside of the study site. All the requirements of the data protection legislation will apply to the processing of your personal data. If you would like more information about how your data will be processed under the terms of UK data protection laws please visit the link below:
https://www.sussex.ac.uk/ogs/policies/information/dpa

WHAT SHOULD I DO IF I WANT TO TAKE PART? If after reading the information sheet you would like to participate in the study, you can email the lead researcher Kamilla Abdullayev (kga21@sussex.ac.uk) to confirm your availability. Before starting the focus group, you will be asked to fill out an online survey to give full informed consent and you will not be able to take part in the study until you have done so.

WHAT WILL HAPPEN TO THE RESULTS OF THE RESEARCH STUDY? The results of this study will be passed on to a team of engineers who will create a prototype of a holistic digital monitoring tool to help improve diagnosis of heart disease. The findings will all be anonymised but will collectively inform which features will be included in this device and how it can be effectively implemented into clinical care. The ultimate goal is to improve how heart disease is diagnosed, making it faster and more accurate. We also plan to publish the findings collected from all the focus groups to contribute to the cardiovascular health literature by providing a patient perspective on the diagnostic process. If you would like to receive updates about papers or other activities which arise from this research, let us know in the consent form and we will notify you. Personal data will be destroyed within 10 years of collection, however the anonymised findings of the interviews will be securely stored for future research at the University of Sussex.

WHO IS FUNDING THE RESEARCH? This project is being carried out by researchers and undergraduate students at the University of Sussex, School of Psychology and has been funded by the Engineering and Physical Sciences Research Council (EPSRC).

WHO HAS REVIEWED THE STUDY? This research has been approved by Sciences & Technology Cross-Schools Research Ethics Committee (SCITEC) at the University of Sussex.

The ethical review application number of this study is ER/FM409/2.

CONTACT DETAILS: If you have any concerns relating to this project please contact lead researcher, Kamilla Abdullayev (kga21@sussex.ac.uk) or chief investigator Dr Faith Matcham (f.matcham@sussex.ac.uk) and/or the Chair of the Science and Technology Cross Schools Research Ethics Committee, (crecscitec@sussex.ac.uk.

INSURANCE: The University of Sussex has insurance in place to cover its legal liabilities in respect of this study.

THANK YOU: Thank you for taking the time to read this information sheet carefully before giving consent to participate in this study.

DATE: 31st October 2022

End of Block: ppt type and info sheet

Start of Block: consent info

Q0 The following questions will be asking you to give your informed consent to take part in the study. In order to participate in the study, you will need to answer 'YES' to all of the questions below. Please read each question carefully and ask the researcher any questions if you are unsure. You have the right to withdraw at any point, participation is entirely voluntary.

End of Block: consent info

Start of Block: questions

*

Q1 Please enter your unique identifier code (Last three letters of your last name followed by the last three digits of your phone number). Please do not enter any spaces or capital letters. It should look similar to this: abc123

____

Q2 I consent to taking part in the focus group.

○ YES (1)

Q3 I understand that the focus group discussion will be audio-recorded.

- ○ YES (1)

Q4 I understand that my participation is entirely voluntary, that I can choose to withdraw from the group discussion at any point without having to give a reason and without penalty.

- ○ YES (1)

Q26 I understand that due to the nature of the group discussion that it may not be possible to withdraw my data after taking part.

- ○ YES (1)

Q5 I understand that all the statements that are made in the focus group discussion will be treated by the other participants in the focus group as private and confidential as fas as agreed at the start but that the researchers cannot make that guarantee on others' behalf

- ○ YES (1)

Q6 I understand that my personal data will be used for the purposes of this research study and will be handled in accordance with Data Protection legislation. I understand that the University's Privacy Notice provides further information on how the University uses personal data in its research.

- ○ YES (1)

Q7 I understand that my collected data will be stored in a de-identified way (e.g. using ID numbers not names), and kept separate from other details about me (e.g. from the consent form). Electronic data will be stored securely on a University managed system.

- ◯ YES (1)

Q23 I understand that my identity will remain confidential in any written reports of this research, and that no information I disclose will lead to the identification in those reports of any individual either by the researchers or by any other party, without first obtaining my written permission.

- ◯ YES (1)

Q24 I understand that de-identified data may be made publicly available, for example through Open Science Framework online data repositories, journal publication or at the request of other researchers.

- ◯ YES (1)

Q25 I have read the information sheet, had the opportunity to ask questions and I understand the principles, procedures and possible risks involved.

- ◯ YES (1)

Q8 (Optional) I agree to be contacted by members of this team about future research that may stem from this study.

- ◯ YES (1)
- ◯ NO (2)

Q27 (Optional) I would like to be updated with the results of the study.

○ YES (1)

○ NO (2)

Q9 I agree to take part in the above University of Sussex research project

○ YES (1)

End of Block: questions

Start of Block: GP info

Q13 The following questions will ask you to enter information about your GP. This information will only be used in case of emergency and we will not contact them without notifying you first. If you have any questions about this, please ask the researcher.

Q14 Please enter the name of your GP

____

Q15 Please enter the address of your GP

____

Q16 Please enter the contact number of your GP

____

End of Block: GP info
