# Supplementary material for: Exploring Stakeholder Perspectives on the Barriers and Facilitators of Implementing Digital Technologies for Heart Disease Diagnosis: Qualitative Study
Source: JMIR Cardio. 2025 Mar 5;9:e66464. doi: 10.2196/66464 (PMC11923470; doi:10.2196/66464)
Supplement: Multimedia Appendix 3 [file cardio_v9i1e66464_app3.pdf]

# Digital Twin Demographics

# Survey Flow

**Standard: participant check (3 Questions)**
**Standard: Introduction block (1 Question)**
**Block: Demographics (4 Questions)**
**Standard: DASS-10 (1 Question)**
**Standard: Digital Technology Questions (2 Questions)**
**Standard: Clinician Questions (3 Questions)**
**Standard: Lived Experience Participant Questions (2 Questions)**

Page Break

Start of Block: participant check

Q16 Participant Type

○ Lived experience of heart disease (1)

○ Carer (2)

○ Clinician (4)

---

Q19 Please enter your unique identifier code that was emailed to you by a member of the research team (e.g. HD001 or CL001)

________________________________________________________________

---

Q21 If you have a Prolific ID, please enter it below

________________________________________________________________

End of Block: participant check

Start of Block: Introduction block

Q18 The following questions will be asking you for your demographic information, as well as your experience with digital technologies and your medical background. You do not have to answer any questions you are not comfortable with. All information will remain confidential, so it will not be shared with anyone outside of our research team.

End of Block: Introduction block

Start of Block: Demographics

Q2 Please enter your age in numeric format (e.g. 45)

________________________________________________________________

---

Q3 What gender do you identify with?

- ○ Prefer not to say (1)
- ○ Female (2)
- ○ Male (3)
- ○ Transgender Male (4)
- ○ Transgender Female (5)
- ○ Non-Binary (6)
- ○ Other (7)

Q4 What is your ethnic group or background?

- ○ Prefer not to say (1)
- ○ English/Welsh/Scottish/Northern Irish/British (2)
- ○ Irish (3)
- ○ Gypsy or Irish Traveller (4)
- ○ Any other White Background (5)
- ○ White and Black Caribbean (6)
- ○ White and Black African (7)
- ○ White and Asian (8)
- ○ Any other Mixed/Multiple Ethnic Background (9)
- ○ Indian (10)
- ○ Pakistani (11)
- ○ Bangladeshi (12)
- ○ Chinese (13)
- ○ Any other Asian Background (14)
- ○ African (15)
- ○ Caribbean (16)
- ○ Any other Black/African/Caribbean Background (17)
- ○ Arab (18)
- ○ Any other ethnic group (19)

---

Q5 Which of the following represents your household's annual gross income (income from all sources before deductions for income tax and national insurance)?

- ○ Prefer not to say (1)
- ○ Less than £15,000 (2)
- ○ £15,000-£24,000 (3)
- ○ £24,000-£40,000 (4)
- ○ £40,000-£55,000 (5)
- ○ More than £55,000 (6)

End of Block: Demographics

---

Start of Block: DASS-10

Q6 Please read each statement and select the statement that indicates how much each item has applied to you over the PAST WEEK.
There are no right or wrong answers. Do not spend too much time on any statement.

| | Never (0) (1) | Sometimes (1) (2) | Often (2) (3) | Almost Always (3) (4) |
|---|---|---|---|---|
| I felt I was close to panic (1) | ○ | ○ | ○ | ○ |
| I found it difficult to work up the initiative to do things (2) | ○ | ○ | ○ | ○ |
| I felt downhearted and blue (3) | ○ | ○ | ○ | ○ |
| I was intolerant of anything that kept me from getting on with what I was doing (4) | ○ | ○ | ○ | ○ |
| I felt that I had nothing to look forward to (5) | ○ | ○ | ○ | ○ |
| I felt scared without any good reason (6) | ○ | ○ | ○ | ○ |
| I tended to over react to situations (7) | ○ | ○ | ○ | ○ |
| I was worried about situations in which I might make a fool of myself (8) | ○ | ○ | ○ | ○ |
| I found it difficult to relax (9) | ○ | ○ | ○ | ○ |
| I couldn't seem to experience any positive feelings at all (10) | ○ | ○ | ○ | ○ |

End of Block: DASS-10

Start of Block: Digital Technology Questions

Q7 Do you currently use any of the following technology? (You can click as many as you need to)

- ☐ TV (1)
- ☐ Mobile Phone (2)
- ☐ Tablet/iPad (3)
- ☐ Desktop Computer (4)
- ☐ Laptop (5)
- ☐ Smart Watch (6)
- ☐ Other, please specify below (7)

________________________________________________

Q8 How confident do you feel in your knowledge of using digital technologies?

- ○ Not confident at all (1)
- ○ Somewhat Confident (2)
- ○ Fairly Confident (3)
- ○ Confident (4)
- ○ Very Confident (5)

End of Block: Digital Technology Questions

Start of Block: Clinician Questions

*Display This Question:*

*If Participant Type = Clinician*

Q10A How many years have passed since you received your medical qualification?

- ◯ less than 5 years (1)
- ◯ 5-10 years (2)
- ◯ 11-15years (3)
- ◯ 15-20 years (4)
- ◯ 20+ years (5)

---

*Display This Question:*

*If Participant Type = Clinician*

Q11A Please select the healthcare service you work in

- ◯ Primary Care (1)
- ◯ Secondary care (2)

---

*Display This Question:*

*If Participant Type = Clinician*

Q17 Please specify your clinical job title

________________________________________________________________

**End of Block: Clinician Questions**

---

**Start of Block: Lived Experience Participant Questions**

*Display This Question:*

*If Participant Type = Lived experience of heart disease*

Q11B Please indicate which of the following heart diseases apply to you

- ☐ Prefer not to say (1)
- ☐ Heart Attack (Myocardial Infarction) (2)
- ☐ Heart Valve Disease (3)
- ☐ Heart Failure (4)
- ☐ Atrial Fibrillation (5)
- ☐ Pacemaker or Defibrillator Implanted (6)
- ☐ High Blood Pressure (7)
- ☐ Angina (8)
- ☐ Other, please specify (9) ________________________________________________

Page Break

*Display This Question:*

*If Participant Type = Lived experience of heart disease*

Q10B Apart from your heart trouble, do you have any other long-standing illnesses or disabilities? If yes, please indicate which of the following applies

- ☐ Prefer not to say (1)
- ☐ Asthma (2)
- ☐ Chronic Bronchitis (3)
- ☐ Other Chest Trouble (4)
- ☐ Diabetes (5)
- ☐ Depression (6)
- ☐ Stomach or Digestive Disorder (7)
- ☐ Liver Trouble (8)
- ☐ Kidney Trouble (9)
- ☐ Rheumatoid Arthritis (10)
- ☐ Osteoarthritis (11)
- ☐ Cancer (12)
- ☐ High Blood Pressure (13)
- ☐ Multiple Sclerosis (14)
- ☐ Epilepsy/Fits (15)
- ☐ Stroke (16)
- ☐ Other neurological trouble (17)

- [ ] Migraine (18)
- [ ] Back Trouble (19)
- [ ] Other, please specify below (20)

________________________________________________

- [ ] None of the above (21)

**End of Block: Lived Experience Participant Questions**

---
