# Supplementary material for: Exploring Stakeholder Perspectives on the Barriers and Facilitators of Implementing Digital Technologies for Heart Disease Diagnosis: Qualitative Study
Source: JMIR Cardio. 2025 Mar 5;9:e66464. doi: 10.2196/66464 (PMC11923470; doi:10.2196/66464)
Supplement: Multimedia Appendix 4 [file cardio_v9i1e66464_app4.docx]

| **Themes and Subthemes** | **Technology-specific recommendation** | **System-level recommendations** |
| --- | --- | --- |
| **Theme 1: Resource-related barriers** | | |
| Clinician Workload | • Intuitive clinician interface  • Integrated with existing IT systems.  • Presents summary, not raw, data.  • AI^a^ -generated interpretation | • Training of early-career clinicians  • System-wide support to reduce clinician workload to accommodate new care pathways  • Faster regulatory pathway for LLMs^b^ in health care |
| Cost implications | • Low unit cost  • Favorable business model | • Reimbursement or tariff that covers cost to patient or health care system.  • Reinvestment of cost savings |
| Systemic Barriers | • Adaptation of existing familiar technologies  • Frictionless integration with IT | • Providing support, training, and helpdesk functions for users  • Investment in underlying IT infrastructure |
| **Theme 2: technology-related barriers** | | |
| Complexity of technology | • Intuitive use devices  • Frictionless to user  • Incremental design | • User education  • Cross-familiarization from non–health care sectors  • Road map of incremental progress |
| Data security and Privacy | • Privacy-by-design  • Appropriate accreditation and cybersecurity safeguards | • Transparency over data use and access |
| Safety concerns | • Safety-by-design | • High-quality clinical trials providing evidence of safety.  • Education and training of clinicians and patients |
| Unreliability | • Validated performance against intended purpose  • Postmarket surveillance and audit  • Active reporting of bugs or issues | • Clinical testing in research  • Pilot implementation and rapid design iteration |
| **Theme 3: user-related barriers** | | |
| Negative user attitudes | • Iterative co-design to maximize acceptability  • Customizability  • Data opt-outs  • Proof of effectiveness | • Nontechnological options available  • Pre-implementation engagement and dialogue |
| Worsening care experience | • Low workload for user  • Clear time or effort savings for the user  • Replacement of burdensome, not positive, aspects of health care | • Routine assessment of user experience and patient outcomes |
| Individual-level variation | • Plurality of options available (including current standard of-care)  • Mitigation of technology biases (against disability and skin tone) | • Acceptance of plurality, not monopoly |
| **Theme 4: resource-related facilitators** | | |
| Cost-effectiveness | • Robust cost-effectiveness evidence | • Benefits directed to payers  • Longer-term budgetary planning |
| Efficiency | • Should reduce overall time spent interacting in health care  • Should improve outcomes (patient satisfaction, clinical outcomes, and cost-effectiveness) | • Identify other areas to simultaneously reduce or deprioritize to avoid adding to existing workload |
| Setup support | • Implement wraparound  technical and nontechnical  support | • Wider education  • Peer-to-peer support |
| **Theme 5: technology-related facilitators** | | |
| Accuracy and reliability | • Verification and validation  • Openly accessible data on accuracy and reliability • Inbuilt QC^c^ and abnormal data flagging functions | • Interoperable data standards  • Appropriate regulatory framework that balances the need for innovation against the need for safety and accuracy |
| Adequate safety considerations | • Clear processes for data flows, monitoring, or escalation | • Education and training of clinicians and patients on correct use and potential safety issues |
| Ease of use | • Intuitive, simple use  • Adaptation of existing hardware or processes | • Comprehensive training or support for users |
| Patient right to data | • Providing data to both user and clinician | • Improved ability for patients to access their own health care data |
| Personalization | • Hardware and interfaces customizable to clinician and user preference  • Provision of personalized data reports | • Procurement of adaptable, customizable technologies, not “one-size-fits-all” |
| **Theme 6: user-related facilitators** | | |
| Adapting to individual characteristics | • Adaptable to different types of users | • Tailored training and support to meet different levels of technological needs |
| Positive user attitudes | • Adopt best-practice design principles from successful existing technologies | • Patient champions and success stories |
| Improving quality of care experience | • Design to address current limitations of care experience | • Embedded quantification of care experience within clinical studies and routine use |

^a^AI: artificial intelligence. ^b^LLM: large language model. ^c^QC: quality control.
